# Proteogenomic characterisation of primary oral cancer unveils extracellular matrix remodelling and immunosuppressive microenvironment linked to lymph node metastasis

**DOI:** 10.1002/ctm2.70261

**Published:** 2025-03-04

**Authors:** Yu Liu, Zhenyu Yang, Jingya Jane Pu, Jie Zhong, Ui‐Soon Khoo, Yu‐Xiong Su, Gao Zhang

**Affiliations:** ^1^ Department of Thoracic Surgery/Institute of Thoracic Oncology West China Hospital Sichuan University Chengdu China; ^2^ Faculty of Dentistry The University of Hong Kong Hong Kong Hong Kong; ^3^ Department of Pathology School of Clinical Medicine The University of Hong Kong Hong Kong Hong Kong

**Keywords:** lymph node metastasis, oral squamous cell carcinoma, single‐cell analysis

## Abstract

Oral squamous cell carcinoma (OSCC) is an increasingly prevalent malignancy worldwide. This study aims to understand molecular alterations associated with lymph node metastasis of OSCC in order to improve treatment strategies. We analysed a cohort of 46 patients with primary OSCC, including 10 with lymph node metastasis and 36 without. Using a comprehensive multi‐omics approach – encompassing genomic, transcriptomic, proteomic, epigenetic, single‐cell, and spatial analyses – we integrated data to delineate the molecular landscape of OSCC in the context of lymph node metastasis. Our genomic analysis identified significant mutations in key genes within the MAPK, TGF‐β and WNT signalling pathways, which are essential for tumour development. The proteogenomic analysis highlighted pathways critical for lymph node dissemination and factors contributing to an immunosuppressive tumour microenvironment. Elevated levels of POSTN were found to reorganise the extracellular matrix (ECM), interact with TGF‐β, disrupt cell cycle regulation and suppress the immune response by reducing VCAM1 activity. Integrated analyses of single‐cell and spatial transcriptome data revealed that cancer‐associated fibroblasts (CAFs) secrete TGF‐β1/2, promoting cancer cell metastasis through epithelial–mesenchymal transition (EMT). Our integrated multi‐omics analysis provides a detailed understanding of molecular mechanisms driving lymph node metastasis of OSCC. These insights could lead to more precise diagnostics and targeted treatments.

This article is protected by copyright. All rights reserved

## 1 INTRODUCTION

Oral squamous cell carcinoma (OSCC) is the predominant oral malignancy, representing 80–90% of all oral cancers.^1^ The incidence of OSCC is rising, driven by factors such as smoking, alcohol consumption, betel quid chewing, human papillomavirus (HPV) infection, nutritional deficiencies, immune dysregulation, and genetic modifications.^2^, ^3^ These factors lead to genetic mutations, epigenetic changes, and an imbalanced microenvironment, contributing to the initiation and progression of OSCC.^4^ Clinically, OSCC is often diagnosed at advanced stages, posing challenges for treatment and management, with high recurrence rates and poor survival outcomes.^5^ Lymph node metastasis (LNM) is a major determinant of poor prognosis in OSCC, yet its molecular mechanisms remain poorly understood.

Despite advances in surgical and radiation therapies, the prognosis for OSCC patients remains poor due to the high incidence of lymph node metastasis (LNM).^6^ Clinically, LNM is particularly challenging to detect in OSCC, as it may not be apparent in some cases and can only be detected through imaging or biopsy.^5^ Moreover, LNM drastically reduces the 5‐year survival rate from approximately 90% to about 40–50% and increases the likelihood of distant metastasis, particularly to the lungs, bones, and liver.^7^, ^8^, ^9^ However, the molecular drivers of LNM, including the role of the tumour microenvironment (TME) and key signalling pathways, remain poorly characterised. Hence, identifying the molecular mechanisms and cellular transformations associated with LNM in OSCC is crucial for improving prognosis and reducing the risk of distant metastasis or tumour recurrence.

As a malignant epithelial tumour, OSCC presented highly metastatic potential due to the activation of the epithelial–mesenchymal transition (EMT).^10^ EMT is a process during which cancer cells undergo phenotypic changes that include the loss of intercellular adhesion and apical‐basal polarity.^11^ TGFβ is a key inducer of EMT, which leads to the disruption of epithelial‐cell junctions, switches in cell elongation, and enhanced motility for directed migration and invasion through the extracellular matrix (ECM).^12^ The alterations in the ECM can activate cell surface receptors (e.g., integrins) and initiate intracellular signalling cascades that promote EMT in cancer cells, ultimately contributing to cancer metastasis and invasion.^13^ Despite these insights, the interplay between EMT, TGF‐β signalling, and ECM remodelling in the context of LNM remains poorly understood.

Numerous studies have explored biomarkers associated with OSCC progression through single‐cell resolution, genomic profiling, and experimental analysis. Researchers have identified biomarkers such as *Cyclin L1*, *MMP10*, vimentin, *ROS 1*, *FGF8*, and *ZEB1*, and key pathways like NF‐κB, ERK‐STAT1/3, and EGFR.^14^, ^15^, ^16^, ^17^, ^18^, ^19^, ^20^, ^21^, ^22^ Additionally, pivotal cellular subtypes such as *CXCL8*‐expressing cancer‐associated fibroblasts, *LAIR2*‐expressing Treg cells, and *SPP1+* macrophages have been implicated in OSCC metastasis.^15^, ^23^, ^24^ In addition, the dynamic immune microenvironment alterations may contribute to the development of LNM in OSCC, including an upregulation of PD‐L1 expression on dendritic cells and an increase in the number of naive and quiescent CD4+ T cells, which have been linked to immune suppression.^25^, ^26^ Overall, these discoveries enhance our understanding of LNM in OSCC and provide a foundation for developing novel diagnostic and therapeutic strategies to improve patient outcomes. However, a comprehensive understanding of how these molecular and cellular changes collectively drive LNM is still lacking.

Several key scientific issues remain unresolved regarding LNM in OSCC progression. The influence of genomic mutations on pathways and biological processes facilitating cancer cell proliferation and metastasis is not fully understood. Additionally, the transformation and aberrant crosstalk among the most critical cell types in the OSCC tumour microenvironment, including tumour cells, cancer‐associated fibroblasts (CAFs), endothelial cells, and immune cells, have not been elucidated. Furthermore, the cooperation of genes and pathways with cellular communications in reconstructing the tumour microenvironment is unclear, and key therapeutic targets for LNM have yet to be identified. To address these gaps, we conducted a multi‐omics analysis to systematically characterise the molecular and cellular mechanisms driving LNM in OSCC.

Currently, treatment for OSCC with LNM often involves a combination of surgery, radiation therapy, chemotherapy, and concurrent systemic therapy (targeted therapy and immunotherapy).^27^ However, the optimal treatment approach depends on factors such as the extent of lymph node involvement and patient health status. Furthermore, the efficacy of these therapies is limited by intratumoural and intercellular heterogeneity, as well as complex cell interactions within the tumour microenvironment (TME).^28^ Our study aims to address these limitations by identifying key molecular drivers of LNM, which could enable early detection of high‐risk patients and the development of targeted therapies to improve outcomes. By uncovering novel biomarkers and therapeutic targets, our research provides a foundation for more precise and effective treatment strategies for OSCC patients with LNM.

Next‐generation sequencing (NGS) has significantly enhanced our understanding of the heterogeneity and evolution of different malignancies. Bulk tumour genomics, proteomics, and metabolomics offer paradigms for identifying disease‐related biomarkers and potential molecular targets, advancing our comprehension of malignant transformation and therapeutic strategies.^29^, ^30^, ^31^ However, during tumour development and lymph node dissemination, OSCC exhibits aberrant cellular communications and tumour microenvironment remodelling, which traditional bulk tumour analyses cannot fully capture.^32^, ^33^, ^34^ Single‐cell and spatial transcriptomics (ST) have emerged as powerful techniques for capturing transcriptomes with spatial resolution and monitoring target cell attributes during disease development stages.^35^, ^36^

In this study, we assembled a cohort of primary OSCC with and without lymph node metastasis (pLN+ and pLN–) and performed multi‐omics, single‐cell, and spatial analyses to delineate the mechanisms associated with LNM in OSCC. Our comprehensive multi‐omics analysis illuminates the genomic landscape of pLN+ and pLN– OSCC and characterises the pLN+ OSCC as an ‘immune‐suppressive’ subtype. Proteogenomics unveiled tumour environment during LNM dissemination may be fostered by elevated POSTN, potentially inducing ECM reorganisation that interacts with TGF‐β and disrupts cell cycle regulation to suppress the immune response. Moreover, single‐cell and spatial transcriptome analysis indicated that CAFs secrete TGF‐β1/2, activating the TGF‐β pathway in cancer cells, and subsequently promoting them metastasis through epithelial–mesenchymal transition (EMT). By integrating multi‐omics, single‐cell, and spatial analyses, our study bridges the critical gap in understanding the molecular mechanisms underlying LNM in OSCC, particularly the roles of ECM remodelling, TGF‐β signalling, and cellular interactions in the TME during metastatic progression. These insights enhance our understanding of OSCC progression and highlight potential targets for novel therapeutic strategies.

## 2 RESULTS

### 2.1 Patient cohorts and multi‐omics data analysis

Our goal was to investigate the proteogenomic landscape of lymph node (LN) metastasis in primary OSCC. Toward that goal, we retrospectively assembled a cohort of 50 patients who underwent surgical resection of primary tumours and lymph node dissections between July 2016 and December 2019 at Queen Mary Hospital (Hong Kong). After rigorous quality control, we eventually included 46 patients in this study (Figure S1A). Their detailed demographics and clinicopathological features were presented in Table S1. This cohort was divided into two groups, including 10 patients with LNM (pLN+) and 36 without (pLN–). Gender distribution was relatively balanced (45% males in pLN+ vs. 54% males in pLN–). The majority were aged between 50 and 69 years old (6 in pLN+ vs. 20 in pLN‐), non‐smokers (9 in pLN+ vs. 26 in pLN‐), and non‐drinkers (8 in pLN+ vs. 30 in pLN‐). Predominantly, tumours originated from the upper and lower gingiva (50% both in pLN+ and pLN–) (Table 1). Statistical analyses revealed no significant differences in most clinicopathological features between these two groups (*p* > .05), except for pathology staging (*p* < .05). All patients with LNM were diagnosed in advanced stages (stage III/IV) (AJCC 8th edition), confirming that there is an association between late‐stage presentation and poor prognosis.^37^, ^38^ Kaplan–Meier survival analysis highlighted significantly better outcomes for patients without LNM (pLN–), which was also corroborated by the analysis of an independent TCGA cohort of 92 patients with OSCC, including 41 pLN– and 51 pLN+ (Figure S2B and C **)**.^39^

**TABLE 1:** Clinicopathological characteristics of recruited patients.

Features	Primary OSCC with lymph node metastases (pLN+) (*n* = 10, 22%)	Primary OSCC without lymph node metastases (pLN–) (*n* = 36, 78%)	Overall cohort (*n* = 46)	*p* value
Sex				
Male, *n* (%)	4 (40%)	20 (56%)	24 (52%)	.4839
Female, *n* (%)	6 (60%)	16 (44%)	22 (48%)	
Age (years)				
<50, *n* (%)	1 (10%)	9 (25%)	10 (22%)	.5661
50–69, *n* (%)	6 (60%)	20 (56%)	26 (57%)	
≥70, *n* (%)	3 (30%)	7 (19%)	10 (22%)	
Smoking				
Yes, *n* (%)	1 (10%)	10 (28%)	11 (24%)	.4103
No, *n* (%)	9 (90%)	26 (72%)	35 (76%)	
Alcohol intake				
Yes, *n* (%)	2 (20%)	6 (17%)	8 (17%)	1
No, *n* (%)	8 (80%)	30 (83%)	38 (83%)	
Subsites				
Buccal mucosa, *n* (%)	3 (30%)	4 (11%)	7 (15%)	.7192
Oral tongue, *n* (%)	2 (20%)	10 (28%)	12 (26%)	
Upper and lower gingiva, *n* (%)	5 (50%)	18 (50%)	23 (50%)	
Floor of mouth, *n* (%)	0 (0%)	2 (6%)	2 (4%)	
Hard palate, *n* (%)	0 (0%)	1 (3%)	1 (2%)	
Retromolar trigone, *n* (%)	0 (0%)	1 (3%)	1 (2%)	
Pathology staging (AJCC 8th)				
I, *n* (%)	0 (0%)	8 (22%)	8 (17%)	.00756
II, *n* (%)	0 (0%)	10 (28%)	10 (22%)	
III, *n* (%)	1 (10%)	7 (19%)	8 (17%)	
IV, *n* (%)	9 (90%)	11 (31%)	20 (44%)	

**FIGURE 1:**
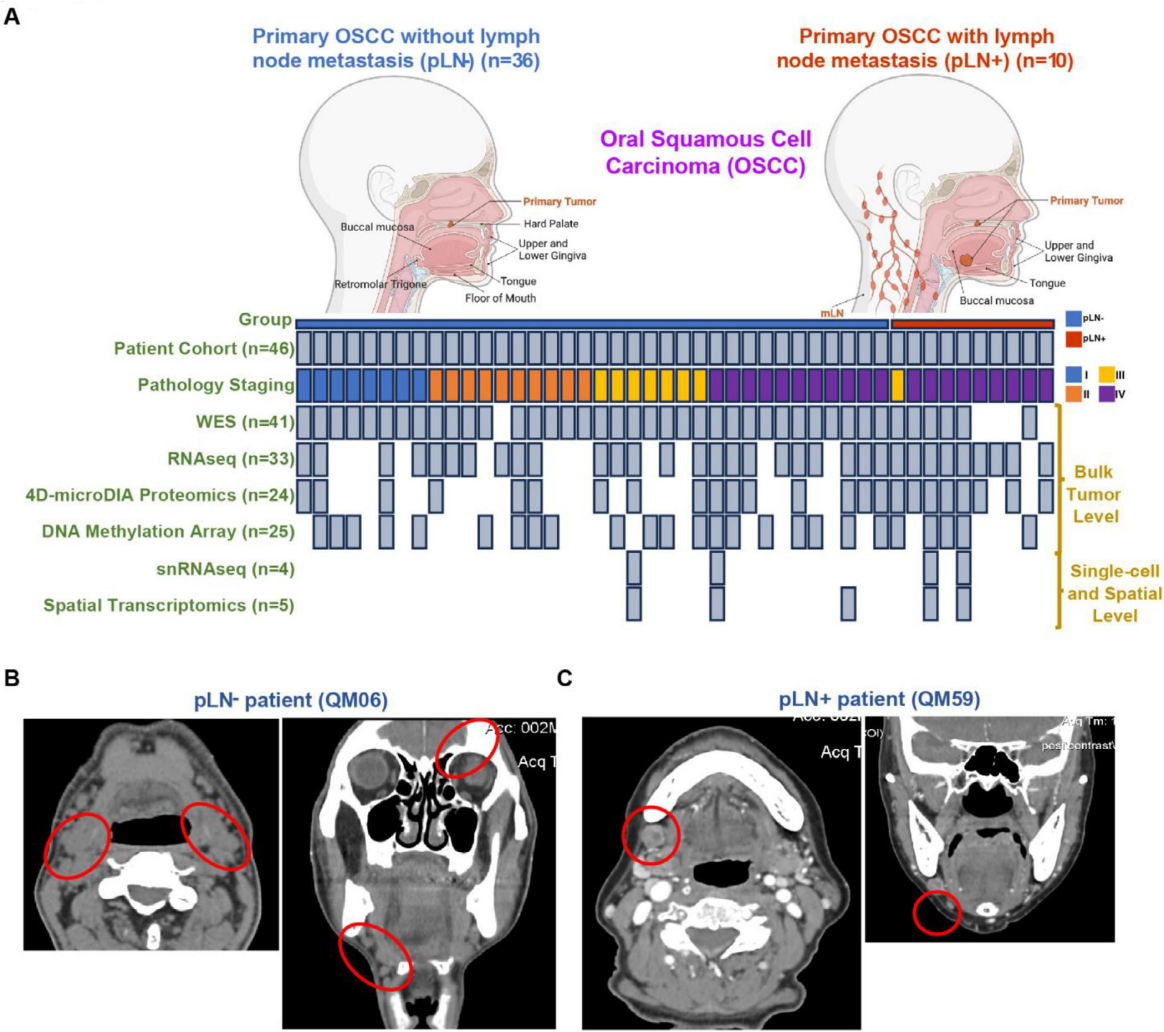
Patient cohort, overview of patients and samples, and experimental design. (A) Schematic summary of two patient cohorts: pLN– (*n* = 36, left) and pLN+ (*n* = 10, right), along with the experimental platforms including WES, RNAseq, 4D‐microDIA proteomics, DNA methylation array, snRNAseq, and spatial transcriptomics. Each column represents one patient's tumour sample with profiling information annotated. (B) Representative CT images from patients with squamous cell carcinoma at the mandible without lymph node metastasis in two views (left: axial, right: coronal). (**C)** Representative CT images from patients with squamous cell carcinoma at the mandible with lymph node metastasis in two views (left: axial, right: coronal).

We then conducted comprehensive proteogenomic profiling, including whole‐exome sequencing (WES) (*n* = 41), RNA sequencing (RNAseq) (*n* = 33), 4D‐microDIA quantitative proteomics (*n* = 24), DNA methylation arrays (*n* = 25), single‐nuclei RNA sequencing (snRNAseq) (*n* = 4), and spatial transcriptomics (*n* = 5) (Figures 1A and S1A and Table S2 **)**.

We also included two clinical cases as illustrative examples, including a patient, QM06, who is a 67‐year‐old male with non‐metastatic OSCC at the mandible, and a patient, QM59, who is an 83‐year‐old female with metastatic OSCC, also at the mandible. Both CT and subsequent histopathological analysis confirmed the absence and presence of LN metastasis for QM06 and QM59, respectively (Figure 1B and 1C).

In summary, our study assembled a cohort of patients’ clinical samples that were suitable for a thorough molecular characterisation of primary OSCC with and without LNM based on an integrative multi‐omic approach.

### 2.2 Genomic insights into primary OSCC with and without lymph node metastasis

To establish the genomic landscape, we conducted whole‐exome sequencing (WES) of primary OSCC samples without lymph node metastasis (pLN–) (*n* = 35) and with lymph node metastasis (pLN+) (*n* = 6). After processing WES data, we identified an average of 154 somatic mutations in pLN+ samples and 229 in pLN– samples by using Mutect2.^40^ The tumour mutational burden (TMB) indicated a median of 2 mutations per megabase (Mb) for pLN+ and 3 for pLN–, respectively, encompassing single nucleotide polymorphisms (SNPs) and small insertions and deletions (Indels) (Figure S2A **)**.^41^ The variant allele frequency (VAF) analysis showed no significant intra‐tumour heterogeneity (ITH) differences between the groups (Figure S2B).^42^

**FIGURE 2:**
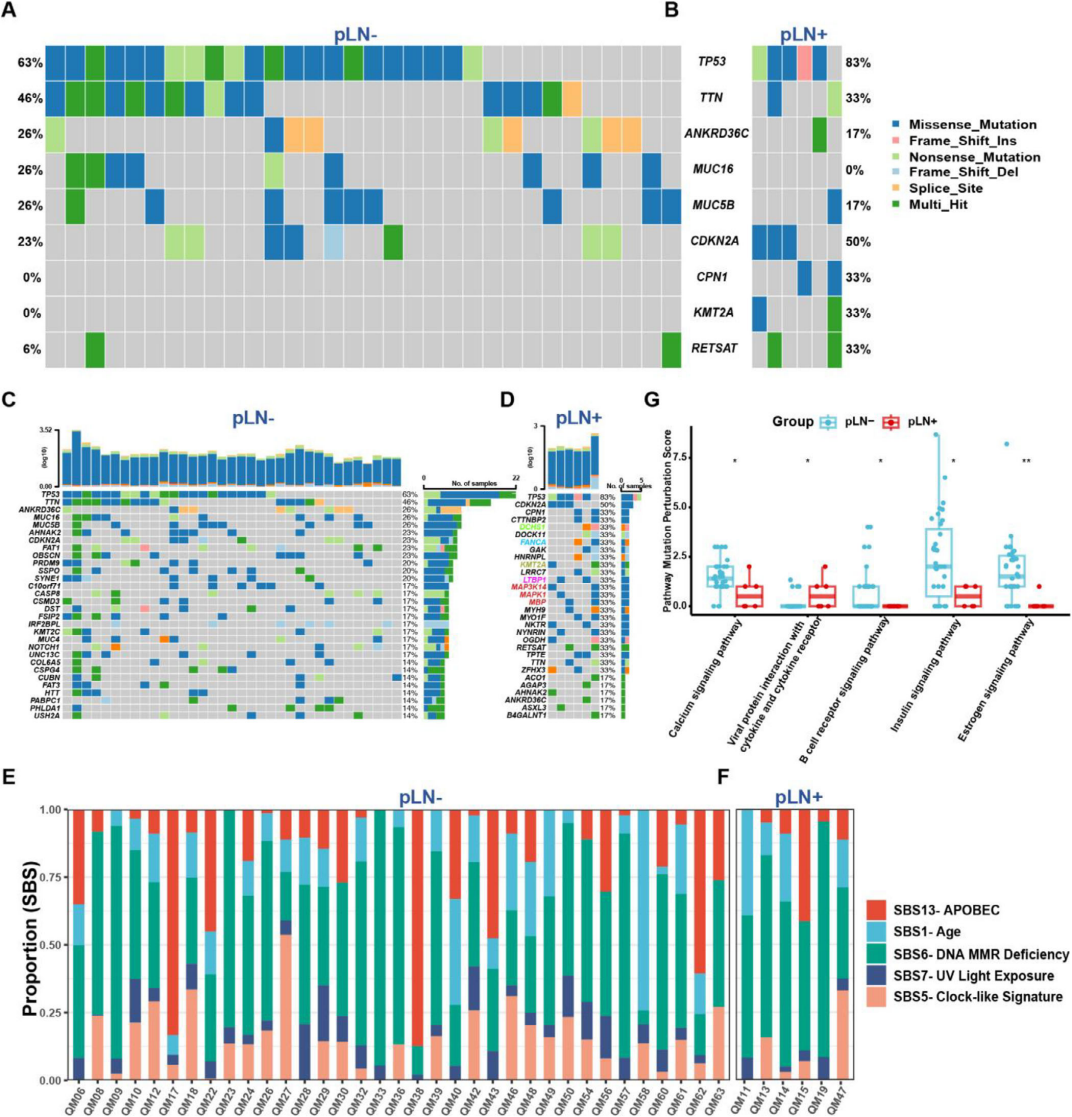
The genomic landscape of pLN– and pLN+ groups. Co‐Oncoplots showing shared and unique mutated genes in pLN– (A) and pLN+ (B) tumour samples from the Queen Mary Hospital (QMH) cohort. Oncoplots showing individual mutated gene patterns in pLN– (C) and pLN+ (D) tumour samples. (E, F) Percentage of mutational signature contribution for each tumour sample. (G) Box plots showing pathway mutation perturbation (PMP) scores for each tumour sample.

Our analysis highlighted alterations in *TP53, TTN, ANKRD36C, MUC5B, CDKN2A*, and *RETSAT* were shared by both groups, underscoring their roles in cell cycle regulation and tumourigenesis for primary OSCC in general.^43^, ^44^, ^45^, ^46^ Unique to pLN– samples were *MUC16*, while *CPN1* and *KMT2A* were distinctive to pLN+ samples (Figure 2A and B). Subsequently, we illustrated the top 30 ranked mutations in both pLN– and pLN+ groups with regard to the mutational frequency of specific genes to further delineate the mutational profiles (Figure 2C and D). Specifically, we identified these mutated genes in the pLN– group were associated with multiple signalling pathways involved in tumourigenesis of HNSCC (*CSDM3, FAT1*, and *NOTCH1*), and cell cycle dysregulation (*CASP8, PRDM9*, and *SYNE1*) (Figure 2C). Likewise, the frequently mutated genes in the pLN+ group implicated the involvement of multiple signalling pathways which are related to tumourigenesis, including (1) WNT signalling pathway (*DCHS1*); (2) DNA repair signalling pathway (*FANCA*); (3) histone modifications signalling pathway (*KMT2A*); (4) TGF‐β signalling pathway (*LTBP1*); and (5) MAPK signalling pathway (*MAP3K14, MAPK1*, and *MBP*) (Figure 2D).

To validate the robustness of our findings and contextualise our cohort within the broader landscape of OSCC genomic alterations, we extended our analysis to an independent cohort of 88 OSCC patients (39 pLN– and 49 pLN+) from TCGA, Specifically, we aimed to determine whether the mutational patterns and key driver genes identified in our cohort, such as TP53, CDKN2A, and TTN, were consistent with those reported in TCGA (Figure S2C and D). Not surprisingly, our analysis revealed a set of similar mutations of *CSMD3, FAT1, NOTCH1, CASP8, CDKN2A, SYNE1* and *MUC16* in the pLN– group (Figure S2E). Likewise, the analysis of the pLN+ group from the TCGA cohort unveiled two frequently mutated genes, *TP53* and *CDKN2A*, followed by *TTN*, *FAT1*, *NOTCH1*, *DNAH5* and *PCLO* (Figure S2F).

The Catalogue of Somatic Mutations in Cancer (COSMIC) analysis revealed no significant differences between both groups with regard to key mutational signatures.^47^ Single base substitution (SBS) mutational signatures SBS 1 (age), SBS 5 (clock‐like signature), SBS 6 (DNA MMR deficiency), SBS 7 (UV light exposure), and SBS 13 (APOBEC activity) were predominant signatures presented in most tumour specimens (Figures 2E and F and S2G).

Next, we employed the Pathway Mutation Perturbation (PMP) score to assess the impact of mutations on specific pathways.^48^ Notably, the *‘Viral Protein Interaction with Cytokine and Cytokine Receptor’* pathway was significantly perturbed in the pLN+ group as compared to the pLN– group, suggesting activation or inhibition of cytokine signalling that possibly affects different aspects of immunity in cancer.^49^ Conversely, four pathways demonstrated significantly higher PMP scores in the pLN– group, including ‘*Calcium Signalling Pathway’, ‘The B Cell Receptor Signalling Pathway’, ‘Insulin Signalling* and *‘Estrogen Signalling Pathway’* (Figure 2G).

Taken together, our results delineated the genomic landscape and identified mutational profiles exhibited by primary OSCC with or without LNM, further offering insights into plausible molecular mechanisms of progression and metastasis of primary OSCC.

### 2.3 Somatic copy number alterations in primary OSCC

To investigate somatic copy number alterations (SCNAs) and their implications for primary OSCC, we conducted the Genomic Identification of Significant Targets in Cancer (GISTIC) analysis.^50^ Specifically, the pLN+ group exhibited 17 arm‐level losses, comprising 7 p‐arms and 10 q‐arms, and 18 peaks of deletion. In contrast, the pLN– group displayed 20 arm‐level gains, including 9 p‐arms and 11 q‐arms, 23 peaks of amplification, and 18 peaks of deletion, indicating a more complex genomic alteration landscape (Figure S3A–D).

Both groups shared all losses and deletions that we identified, with the most significant deletion peak at 18p11.32. Notable regions of alterations included amplifications at 3q29 (featuring genes *SDHA, RPL29, RPL35A*), 11q13.3 (*TIGAR*), and 11q22.2 (*MMP20*), and losses at 3p14.3 (*ADAMTS9*), 7q34 (*BRAF*), and 17q21.2 (*KRT13, CA9*)^51^, ^52^, ^53^, ^54^, ^55^, ^56^, ^57^, ^58^, ^59^, ^60^, ^61^ (Figure S3C and D).

The SCNAs exhibited by both primary OSCC groups elucidate a complex genomic landscape, highlighting the existence of specific genetic alterations that might contribute to the pathogenesis and metastatic potential of primary OSCC.

### 2.4 The analysis of transcriptomic data reveals key insights into the tumourigenesis of pLN+ OSCC

**FIGURE 3:**
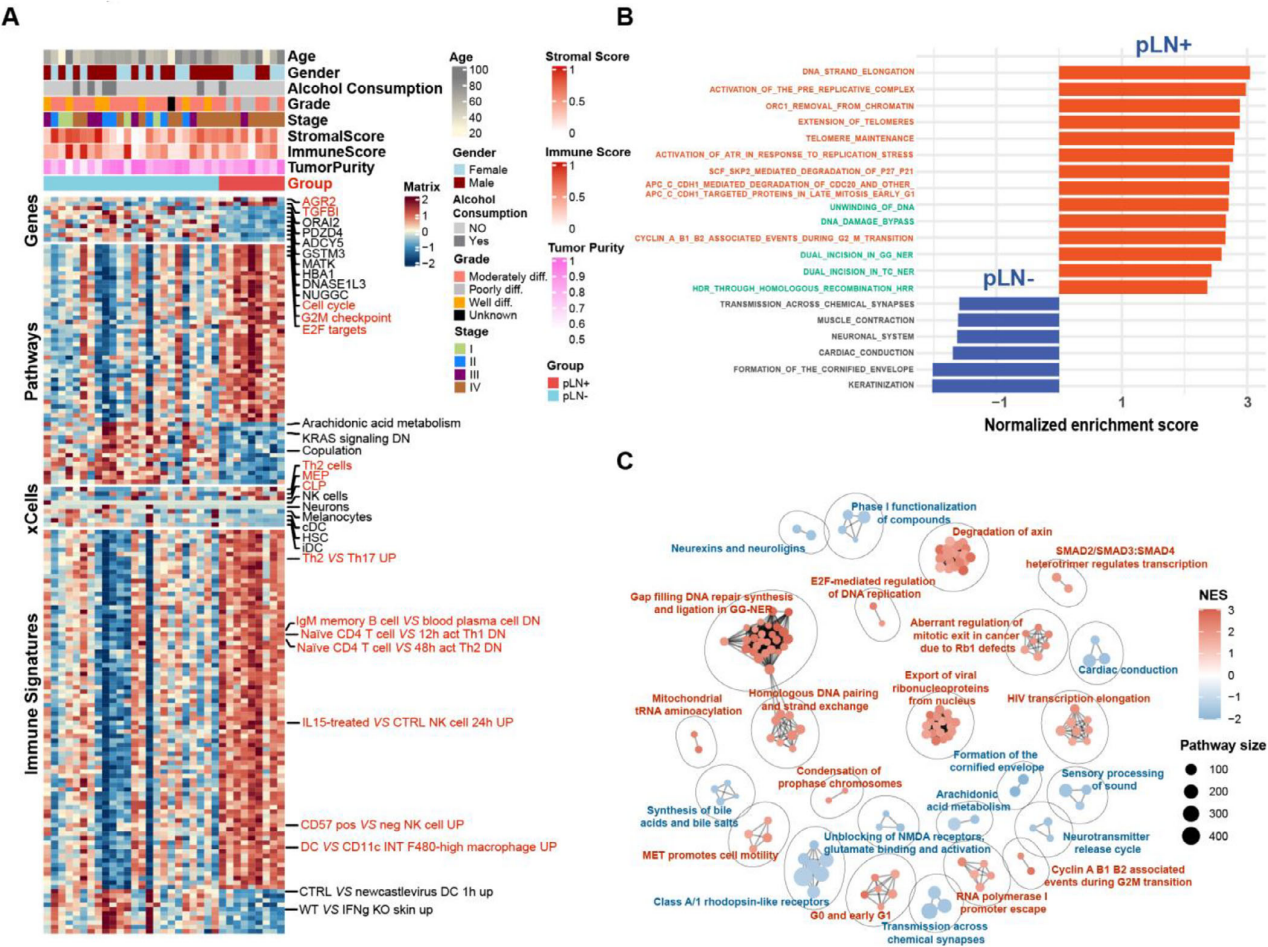
Transcriptomic characterisation of pLN+ and pLN– groups. (A) Heatmap illustrating two groups based on differentially expressed genes, enriched pathways, xCell‐derived cells and immune signatures. Upregulated genes, enriched pathways, abundant immune cells and higher immune scores in pLN+ OSCC are highlighted in red. (B) Gene Set Enrichment Analysis (GSEA) plots of the top 20 pathways (ranked by adj. *p* values) based on the Reactome collection from MSigDB. Red bars towards the right indicate enriched pathways in primary OSCC with lymph node metastasis; blue bars towards the left indicate enriched pathways in primary OSCC without lymph node metastasis. Pathways related to the cell cycle and DNA repair are highlighted in red and green, respectively. (C) Network analysis presenting clusters across enriched pathways in the Reactome dataset based on similarity and connectivity. Clusters enriched in pLN+ OSCC are highlighted in red, and those enriched in pLN– OSCC are highlighted in blue.

Next, we analysed bulk RNA sequencing data derived from 33 tumour samples, including 9 pLN+ and 24 pLN– OSCC. The differentially expressed genes (DEGs) analysis identified that 7 genes were upregulated and 173 were downregulated in pLN+ tumours compared to pLN– (Log2fold Change > 1; adjusted Wald test *p* < .05) (Figures 3A and S4A). Notably, *AGR2*, which significantly influences the EGFR signalling axis and tumour pathogenesis, exhibited the highest increase in the expression level.^62^, ^63^ Other upregulated genes included *TGFβI*, that acts as a downstream regulator of the TGF‐β signalling pathway, influencing epithelial–mesenchymal transition (EMT) and contributing to an immunosuppressive microenvironment in various cancers, including cholangiocarcinoma and ovarian cancer; and *AURKA*, which is a key regulator of the cell cycle.^64^, ^65^, ^66^, ^67^ Subsequently, we performed the Gene Set Enrichment Analysis (GSEA) by using an unbiased computational algorithm (Mann–Whitney–Wilcoxon Gene‐Set Test, MWW‐GST) based on ranked gene lists of DEGs from various gene set collections in the Molecular Signatures Database (MSigDB), including Gene Ontology Biological Process (GO‐BP) (MSigDB C5), Kyoto Encyclopedia of Genes and Genomes (KEGG) (MSigDB C2), Hallmark (MSigDB H), and immunological signature gene sets (MSigDB C7).^68^, ^69^, ^70^ Specifically, pLN+ OSCC showed enrichment of cell cycle pathways, particularly the G2M checkpoint and E2F targets, essential for cell proliferation and DNA replication.^71^, ^72^ In contrast, pLN– OSCC exhibited an enrichment of pathways related to lipid metabolism (Figure 3A). Therefore, to further validate these pathways enriched in the pLN+ OSCC, we interrogated another collection of gene sets, Reactome, and discovered 296 gene sets being significantly enriched (|normalised enrichment score| > 1 and adjusted *p*‐value < .25) (Table S3). This pathway analysis highlighted the involvement of cell cycle and DNA replication pathways in pLN+ OSCC, such as DNA strand elongation and telomere maintenance, alongside DNA repair pathways, including DNA unwinding and homologous recombination, that were critical for proliferation and metastasis of cancer cells (Figure 3B). Conversely, the signalling pathways enriched in the pLN– group, were predominantly associated with keratinisation and muscle system processes. According to these findings, we carried out the functional analysis via aPEAR (Advanced Pathway Enrichment Analysis Representation) algorithm to identify the interconnected clusters based on the similarities between the pathway gene sets and construct a connective network.^73^ Notably, 6 clusters related to cell cycle and DNA repair were prominent in pLN+ OSCC, comprising (1) Gap filling DNA repair synthesis and ligation in GG‐NER; (2) homologous DNA pairing and strand exchange; (3) G0 and early G1; (4) cyclin A/B1/B2 associated events during G2/M transition; (5) E2F‐mediated regulation of DNA replication; and (6) aberrant regulation of mitotic exit in cancer due to Rb1 defects. Furthermore, pathways involved in tumour invasiveness and metastasis were also connected and presented in pLN+ OSCC, including clusters named ‘MET promotes cell motility’ and ‘SMAD2/SMAD3:SMAD4 heterotrimer regulates transcription’ (Figure 3C). Taken together, our results underscore the enhanced proliferative and metastatic potential of pLN+ OSCC, thereby paving the avenue for further elucidating molecular mechanisms and cellular interactions exhibited by pLN+ and pLN– OSCC.

Furthermore, to investigate the difference in immune infiltration between pLN+ and pLN– lesions, we conducted the CIBERSORTx (LM22) analysis of the bulk tumour RNAseq data to estimate the relative abundance of 22 immune cell populations in tumour samples from these two groups (Figure S4B **)**.^74^ The deconvolution analysis of immune cell populations demonstrated significant decreases in plasma cells within the pLN+ phenotype when comparing various cell subtypes. Since the CIBERSORTx analysis mainly focuses on the relative abundance of immune cell populations, we then used xCell and ESTIMATE immune scores to further evaluate the levels of immune cell infiltration. These two methods can provide a more comprehensive view of immune cell presence and overall immune activity in the tumours.^75^, ^76^ Our results demonstrated stronger signatures of T helper 2 cells (TH2), megakaryocyte–erythroid progenitor cells (MEP), and common lymphoid precursor (CLP) in pLN+ OSCC (Figure 3A). Notably, a prior study linked Th2 effector cells with unfavourable outcomes in OSCC due to the expression of *CCR8*.^77^ Correspondingly, via the enrichment analysis within immunological signature gene sets (MSigDB C7), the pLN+ phenotype showcased an upregulation of the Th2‐involved pathway that is *Th2 VS Th 17 UP* (Figure 3A). Importantly, various studies have provided evidence supporting that activation of Th2 may contribute to the establishment of an immune‐suppressive microenvironment, thereby facilitating tumour progression.^78^, ^79^, ^80^, ^81^ In addition, other signatures related to immune suppression are also implied in the pLN+ OSCC, containing but not limited to *IgM memory B cell VS blood plasma cell DN*, *Naïve CD4 T cell VS 48 h act Th2 DN*, *CD57 pos VS neg NK cell UP*. Corresponding to that the TGFBI may contribute to the immunosuppressive microenvironment, we speculated that pLN+ OSCC may adopt a phenotype of ‘immune‐suppressive’ compared with the pLN– OSCC.^82^

To summarise, our analysis of transcriptomic data integrated findings from immune profiling, gene expression, and pathway enrichment. The comprehensive exploration revealed the presence of immune‐suppressive signatures, as well as the enrichment of the cell cycle and DNA repair pathway in the pLN+ phenotype, providing critical insights into the mechanisms underlying cancer cell dissemination.

### 2.5 Characterisation of the proteome network identifies ECM remodelling in pLN+ OSCC

We undertook the four‐dimensional (4D) data‐independent acquisition (DIA) quantitative proteomics approach to profile 24 fresh frozen tumour specimens, including 8 pLN+ and 16 pLN–.^83^ We subsequently identified 56 proteins that were significantly upregulated in pLN+ OSCC, including TGFβI, which correlates with its mRNA elevation (Figure S5A), RPS6KA4, PLAU and PTPN14, which were linked to key signalling pathways like MAPK, cell cycle and WNT.^84^, ^85^, ^86^ The protein enrichment analysis based on the Reactome collection of gene sets highlighted a significant enrichment of pathways related to extracellular matrix (ECM) remodelling in the pLN+ group, notably in ‘Extracellular Matrix Organisation’ and ‘Integrin Cell Surface Interactions’ (Figure S5B and Table S4). Our findings suggested that pLN+ OSCC exhibited an active ECM reorganisation, which was conducive to tumour progression and metastasis.^87^

**FIGURE 4:**
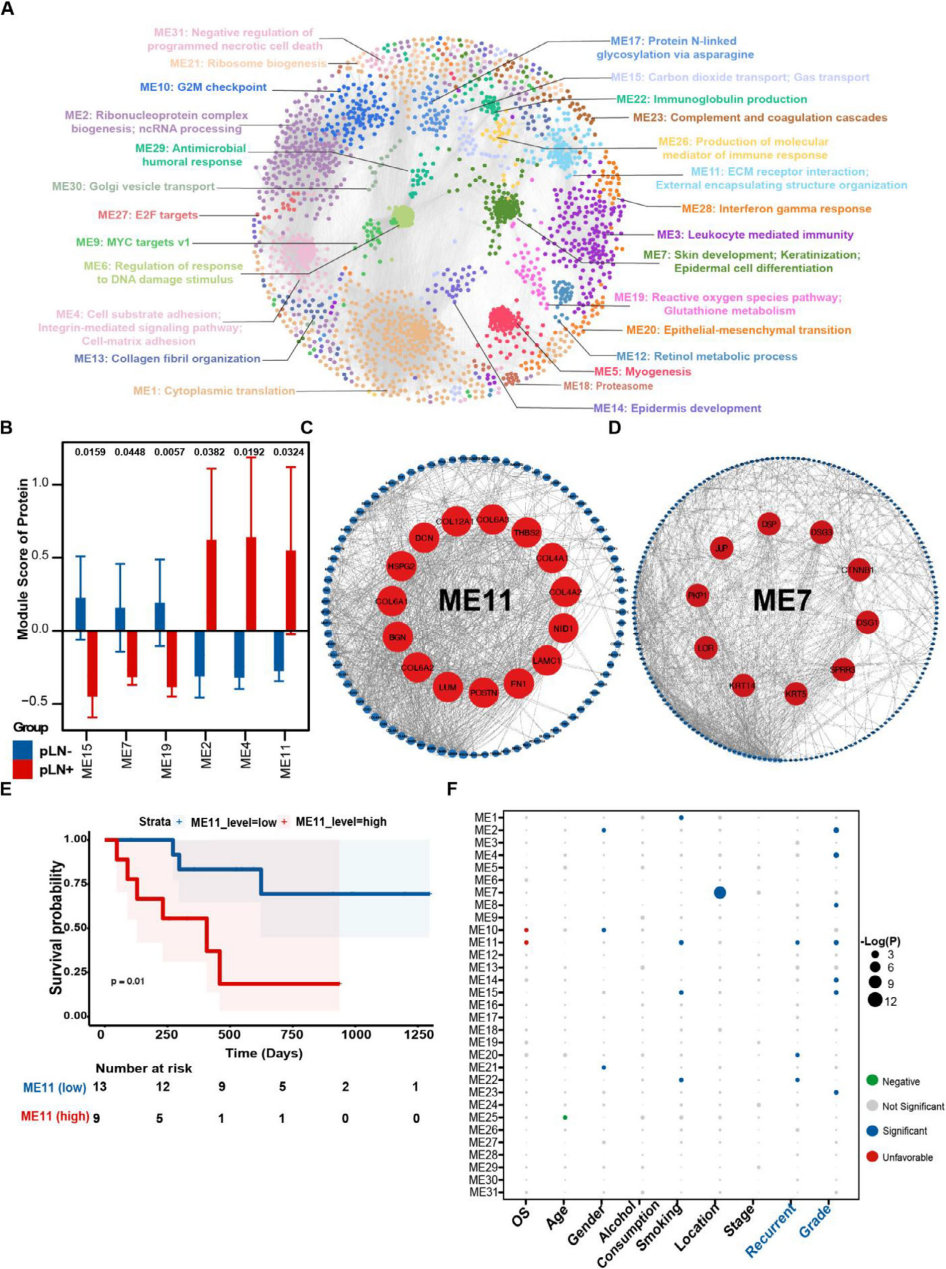
Protein co‐expression network and clinical relevance of functional protein modules. (A) WGCNA identified 30 functional protein modules (ME01–30) enriched in proteomic data for primary OSCC tumour samples. Each network node represents one protein, colour‐coded by different functional modules. (B) Bar plot showing the normalised enrichment score with significant values of the 30 protein modules in pLN+ and pLN– OSCC. *p* Values were calculated using the Mann–Whitney *U*‐test and adjusted using the Benjamini–Hochberg method. Only modules with adjusted *p*  <  .05 are presented. Error bars represent means  ±  S.E. Two‐sided *p* values were calculated. (C) Subnetwork of module 11. The top 15 ranked genes with the highest degree of centrality are highlighted in red and presented in the central circle. (D) Subnetwork of module 7. The top 10 ranked genes with the highest degree of centrality are highlighted in red and presented in the central circle. (E) Kaplan–Meier survival curves comparing OS between patient subgroups stratified by high/low abundance (median cutoff) of ME11. *p* values were calculated using the log‐rank test. (F) Association of the enrichment score of the 30 modules with clinical data. Detailed information regarding the correlation analysis between module scores and clinical data is shown in Methods.

Consistent with the aforementioned findings, we proposed to explore more biological characteristics from a proteome‐wide perspective to investigate potential biomarkers of the dynamic alterations in pLN+ OSCC. To achieve this, we constructed a protein co‐expression network based on protein expression profiling via weighted gene co‐expression network analysis (WGCNA).^88^ A scale‐free network was constructed with scale‐free *R* ^2^ of .8 and soft‐threshold power (β) of 12 as the soft‐threshold values (Figure S5C and D). Subsequently, by employing the ‘cutreeDynamic’ function with minModuleSize = 20, we identified a total of 31 co‐expression modules (ME) (Figure S5E and F). Concurrently, we depicted the modules in a low‐dimensional space and each was associated with unique biological processes and molecular pathways (Figures 4A and S5F). These modules unveiled the essential processes during OSCC development and metastasis, encompassing ME1: Cytoplasmic translation; ME4: Cell substrate adhesion/Cell‐matrix adhesion/; ME9: MYC targets v1; ME 10: G2M checkpoint; ME11: ECM receptor interaction/External encapsulating structure organisation; ME20: Epithelial–mesenchymal transition; ME22:Immunoglobulin production; ME 26: Production of molecular mediator of immune response; and ME27: E2F target. Subsequently, by overlaying the abundance of differentially expressed proteins into the resulting WGCNA network (Figure S5A), we identified 6 modules (adj.*p* values < .05) of significant upregulation in pLN+ OSCC, including ME02, ME04 and ME11, whereas 3 modules exhibited significant elevation in pLN– OSCC, including ME07, ME15 and ME19 (Figure 4B). Specifically, all attributes featured in ME 11 were linked to ECM organisation. Therefore, we designated ME11 as the ECM‐related module (Figures 4A and S5G). In addition, ME 07 was featured with dominant attributes associated with keratinisation, which was collaborated with data derived from the transcriptomic analysis (Figures 4A and S5H). Simultaneously, we calculated the degree centrality (DC) within each module and concentrated on the hub gene possessing top ranked DC scores. The 15 highest‐ranked proteins in ME11 encompassed, but were not limited to, FN1, POSTN, LUM, COL6A2 and BGN, all of which were implied in ECM remodelling (Figure 4C). ME7 showcased the top 10 ranked hub proteins, among which CTNNB1 is a pivotal protein involved in the WNT and EGFR signalling pathways, which has been identified as a therapeutic target for tumourigenesis via a pan‐cancer analysis (Figure 4D).^89^, ^90^, ^91^

Subsequently, we conducted an association analysis to examine the clinical relevance of the identified modules. As expected, a high protein level of ME11 was found to be associated with an unfavourable prognosis of patients with LNM via the Kaplan–Meier analysis (Figure 4E). Furthermore, we calculated the enrichment score of each module with respect to its correlation with clinical data, such as overall survival (OS), age, gender, alcohol consumption, smoking, stage, recurrence, and grade. ME11 was found to be significantly enriched in characteristics that are essential for tumour recurrence and differentiated grade (Figure 4F). Additionally, ME11 was associated with poor OS, which is consistent with the results of the preceding Kaplan–Meier analysis.

Taken together, the analysis of 4D‐DIA quantitative proteomics data not only strengthens the results of our transcriptomic data but also emphasises ECM remodelling as a critical factor that underlies the progression and prognosis of pLN+ OSCC.

### 2.6 Epigenetic insights into pathways underlying tumourigenesis of primary OSCC

Epigenetic regulation is pivotal for the development of primary OSCC, influencing gene expression through mechanisms like DNA methylation and chromatin remodelling.^92^, ^93^, ^94^ To elucidate this, we performed a global DNA methylation analysis of 25 primary OSCC, including 20 pLN– and 5 pLN+ tumours by using the Infinium MethylationEPIC v2.0 BeadChip. This analysis targeted over 935 000 CpG sites, revealing 6763 differentially methylated probes (DMPs) among all the samples. Subsequently, we interrogated the distribution of these DMPs within diverse functional genomic regions and CpG islands. Notably, changes were predominantly found within CpG islands, followed by N‐shore, S‐Shore, N‐Shelf and S‐Shelf (Figure S6A). Upon examing the sites of surrounding genes, we observed that differentially methylated sites were predominantly concentrated at the vicinity of gene bodies, followed by the intergenic region (IGR), transcription start sites (TSS1500, TSS200), 3′UTR, first exons, and 5′UTR (Figure S6A). According to this, the CpG sites with differential methylation were subjected to consensus clustering analysis in order for us to visualise of the extent of DNA methylation between the two groups (Figure S6B).

Among 117 differentially methylated regions (DMRs), a key finding was the demethylation of the *EGFR* gene promoter in pLN+ OSCC (Figure S6C). Prior research has established that this modification likely facilitates an increase in *EGFR* transcription, strengthening the gene's role in activating pathways crucial for the progression of primary OSCC.^95^

Consequently, to elucidate the functional implications of these DNA methylation alterations, we performed the pathway analysis of genes with differentially methylated promoters or bodies. The analysis led to the identification of 64 Gene Ontology Biological Process (GOBP) terms, encompassing multicellular organismal process, multicellular organism development, and stem cell differentiation (Figure S6D). Among these GOBP terms, notably, the top 5 ranked of them were (1) multicellular organismal process; (2) developmental process; (3) anatomical structure development; (4) multicellular organism development; (5) cellular developmental process, which tend to be excessively activated during tumourigenesis of primary OSCC.

In summary, our epigenetic analysis not only identifies a significant demethylation of the *EGFR* promoter in pLN+ OSCC but also highlights the enrichment of pathways critical for tumour progression based on methylated regions. This analysis provides us with valuable insights into how primary OSCC were regulated epigenetically.

### 2.7 Integrated analyses of multi‐omics data highlights key biomarkers of pLN+ OSCC

When attempting the comprehensive data analysis, we conducted a correlative analysis of patient‐matched mRNA and protein data. The results indicated that 81% out of 8375 protein‐coding genes exhibited a positive correlation, among which 26% of genes showed a statistical significance (mean Spearman's coefficient of .23) (Figure S7A).

**FIGURE 5:**
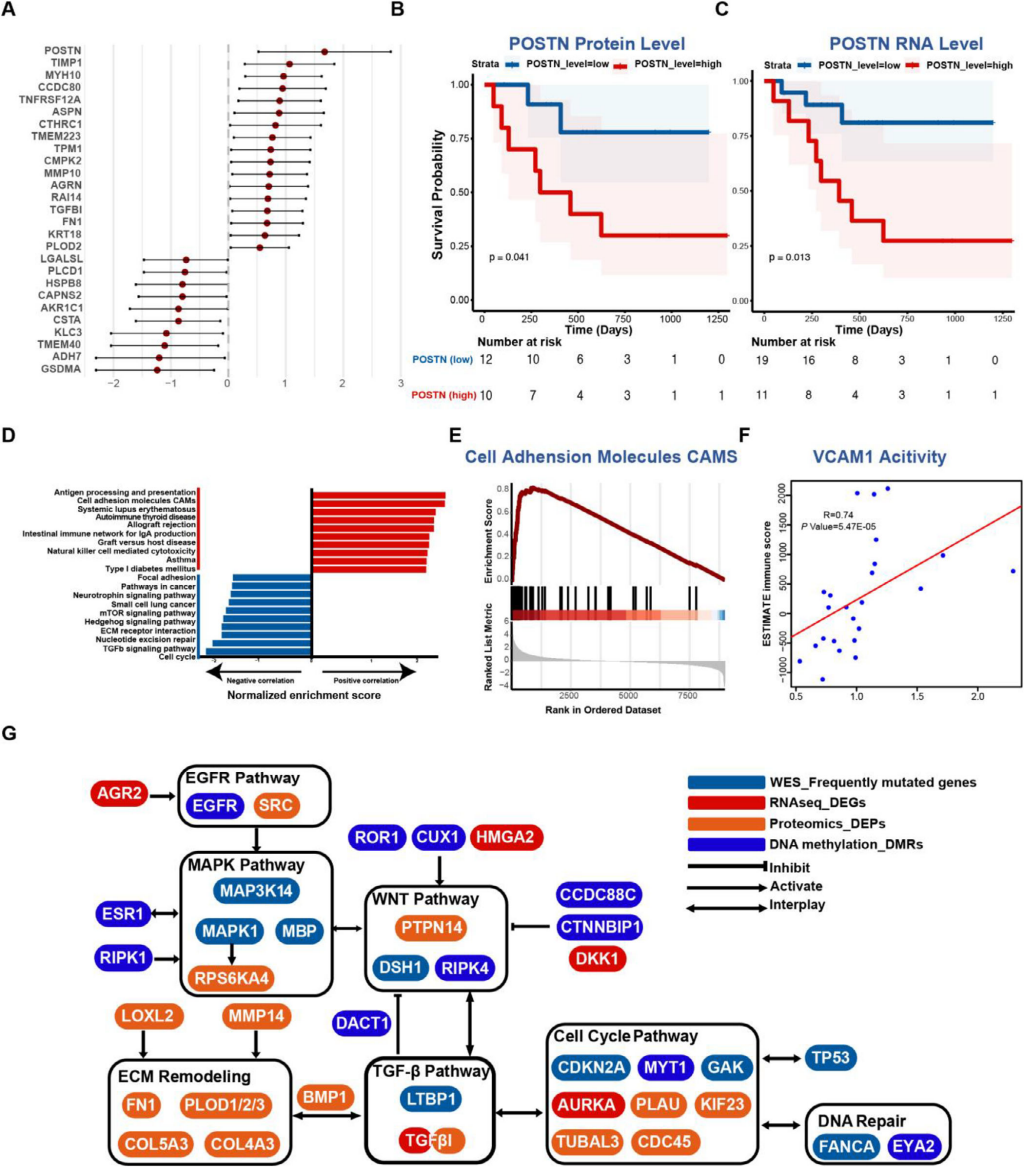
Integrative analysis of bulk tumour sequencing platforms. (A) Forest plot showing the correlated effect of proteins screened from mRNA–protein correlation analysis. 27 proteins are presented with a correlation coefficient greater than .7, adjusted *p* value < .01, and hazard ratio (HR) greater than 2 or less than .5. Kaplan–Meier survival curves comparing OS between patient subgroups stratified by high/low expression (median cutoff) level of POSTN protein (B) and mRNA (C). *p* Values were calculated using the log‐rank test. (D) Bar plot showing normalised enrichment scores for the top KEGG pathways correlated (red) or anti‐correlated (blue) with immune scores. (E) GSEA plot for cell adhesion molecules (CAMs) pathway correlated with immune scores. (F) Scatterplot showing Spearman's correlation of immune scores and VCAM1 activity. (G) Molecular network showing alterations in pLN+ OSCC on genomic, transcriptomic, proteomic and epigenetic levels. Genes identified in distinct platforms are highlighted in different colours.

We then chose the following criteria to select the potential prognostic proteins, including (1) a correlation coefficient greater than .7, (2) adjusted *p* value < .01, and (3) a hazard ratio (HR) greater than 2 for upregulated proteins or HR less than .5 for downregulated proteins (Figure 5A). Of the particular note, POSTN emerged as the protein with the highest positive correlation with prognosis, which was previously ranked as a top‐ranked protein in the ME11 module (Figure 4C). POSTN (periostin) plays a pivotal role in tumour progression by interacting with ECM components and engaging WNT and NOTCH1 signalling pathways.^96^ Consequently, we further evaluated the survival data of the patients from our cohort based on protein and RNA expression levels of POSTN. Our analysis revealed that elevated expression of POSTN adversely correlates with survival for the patients with pLN+ OSCC in our cohort (Figure 5B and C).

Additionally, in order to delve deeper into immune features of pLN+ phenotype, we conducted the correlative analysis by incorporating ESTIMATE immune scores and mRNA–protein data (Figure 5D).^75^ As expected, immunological pathways demonstrated favourable correlations with these proteins, including antigen processing and presentation, autoimmune thyroid disease, intestinal immune network for IgA production, and natural killer cell‐mediated cytotoxicity. However, signalling pathways like ECM receptor interaction, cell cycle, and TGF‐β signalling showed negative correlations with immune scores, suggesting that their upregulation was linked with an immunosuppressive tumour microenvironment of pLN+ OSCC. Remarkably, the cell adhesion molecules (CAMs) pathway exhibited a pronounced positive correlation with immune scores, indicating the proteins in the CAMs family might be associated with immune infiltration (Figure 5D and E). Indeed, VCAM1 (Vascular Cell Adhesion Molecule 1, also referred to as CD106), which is an endothelial cell adhesion molecule, demonstrated a markedly positive correlation with immune infiltration scores (Figure 5F). Interestingly, the overexpression of TGF‐β has been shown to suppress the expression of VCAM‐1 in tumour endothelium, enabling tumour cells to evade immunosurveillance.^97^, ^98^ Collectively, our findings indicate that increases in cell cycle activity, TGF‐β expression, and ECM remodelling may lead to the immune suppression by impeding the activation of VCAM1 expression.

Subsequently, we constructed a comprehensive molecular network that integrates genomic, transcriptomic, proteomic, and epigenetic data of pLN+ OSCC (Figure 5G). This integrated network revealed several pivotal signalling pathways pertinent to tumour development, encompassing EGFR, MAPK, WNT, cell cycle, DNA repair, TGF‐β, and ECM‐remodelling. Intriguingly, these pathways cooperate with each other to promote cancer cell invasion and metastasis, with several genes acting as the connections that work on two or more distinct pathways.

In conclusion, our integrated data analysis delineates a complex network of molecular interactions driving lymph node metastasis of pLN+ OSCC. Importantly, it highlights POSTN and VCAM1 as pivotal nodes influencing ECM remodelling and immune suppression, which interacts with cell cycle dysregulation and TGF‐β expression to promote LN metastasis of primary OSCC.

### 2.8 The analysis of single‐cell RNA sequencing and spatial transcriptome data reveals CAF‐secreted TGFβ1/2 promotes metastasis of pLN+ OSCC

**FIGURE 6:**
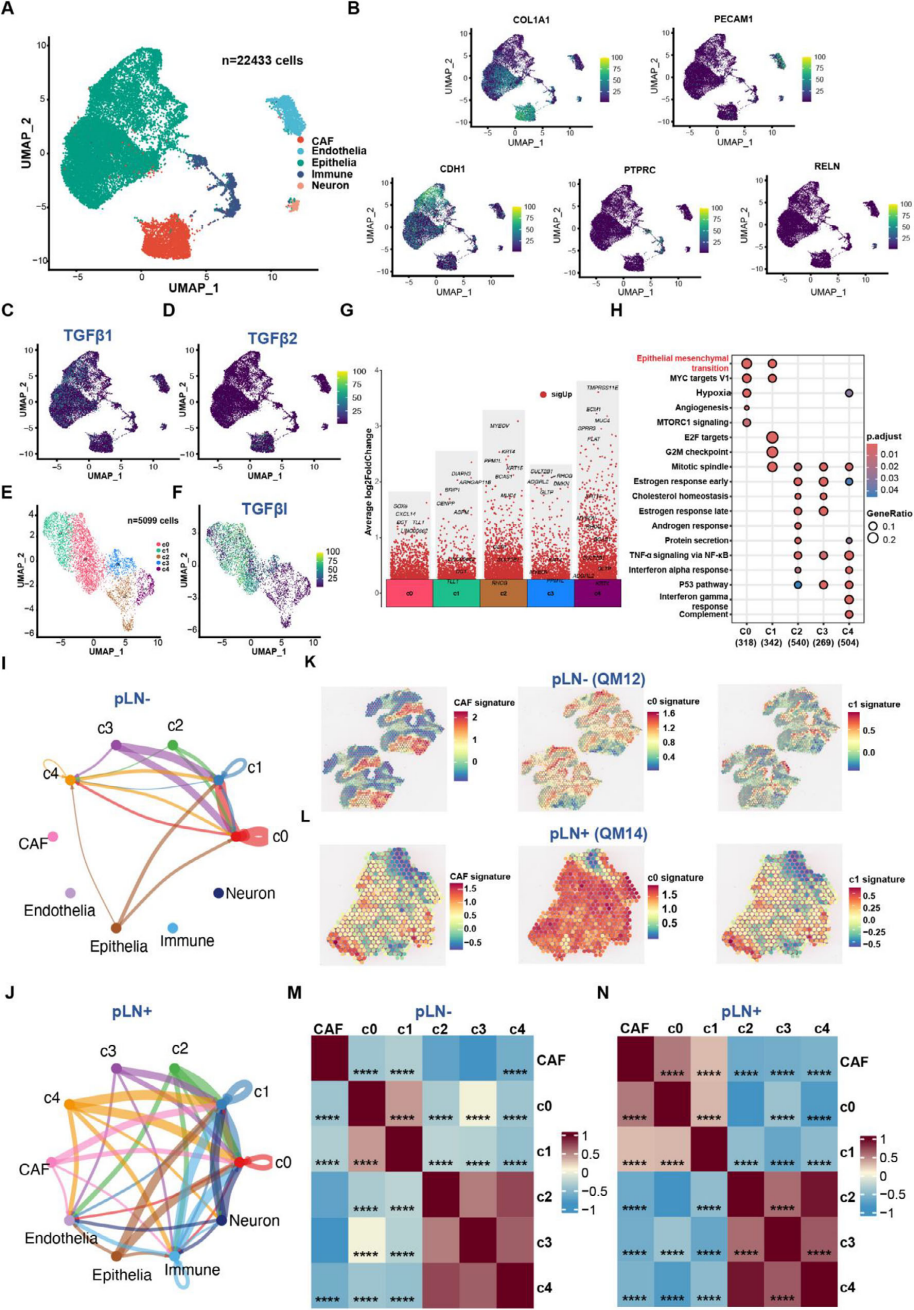
snRNAseq and spatial transcriptomic reveal crosstalk between CAF and cancer cells. (A) UMAP plot of 22 433 cells derived from 4 OSCC patients showing 5 cell subclusters profiled by different colours. (**B)** UMAP atlas showing the expression levels of 5 marker genes. UMAP atlas showing the expression levels of TGF‐β related features, TGF‐β1 (C), TGF‐β2 (D). (E) UMAP plot of 5099 cancer cells scrutinised by CopyKat showing 5 cell subclusters profiled by different colours. (F) UMAP atlas showing the expression levels of TGF‐βI in cancer cells. (G) Differential gene expression analysis showing up‐ and downregulated genes across all 5 clusters. An adjusted *p*‐value < .05 is indicated in red. (H) Bubble plot showing enriched pathways across 5 clusters. The size of the dots represents the fraction of genes involved in the pathway, and the intensity of the colour indicates the adjusted *p*‐value. Circle plot showing intercellular TGF‐β signalling interactions among different cell types and subclusters of cancer cells within pLN– (I) and pLN+ (J) OSCC. Spatial spots showing the expression level of CAF, C0 and C1 signatures in pLN– (K) and pLN+ OSCC (L). Heatmap showing the correlation scores among different cell clusters in pLN– (M) and pLN+ OSCC (N).

Intra‐tumour heterogeneity (ITH) and the tumour microenvironment (TME) are crucial in influencing the progression and metastasis of primary OSCC.^99^, ^100^ To explore this at both single‐cell and spatial levels, we leveraged advanced techniques of single‐nuclei RNA sequencing (snRNAseq) and spatial transcriptomics. Specifically, four primary tumours were subject to snRNAseq, including 2 pLN+ and 2 pLN– OSCC.^101^, ^102^ After quality control, we identified 22 433 cells in total, which were further classified into 10 major clusters (Figure S8A and B). By using markers of specific clusters, we identified five distinct cell types, including cancer‐associated fibroblasts (CAFs) (*COL1A1*), endothelial cells (*PECAM1*), epithelial cells (*CDH1*), immune cells (*PTPRC*), and neurons (*RELN*) (Figure 6A and B). Among them, epithelial cells are commonly regarded as the origins of malignant cells that develop into the OSCC.^32^ In addition, since POSTN is an essential protein for activating the cell cycle/TGF‐β/ECM‐remodelling axis during LNM, we assessed the expression of POSTN and unveiled its higher expression in CAF than other cell types (Figure S8C). Moreover, given that our integrated data analyses at the bulk tumour level revealed the upregulation of the TGF‐β signalling pathway, we focused on genes related to the TGF‐β signalling when analysing snRNAseq data. The analysis established that *TGF‐β1* was highly expressed in CAFs and epithelial cells, whereas *TGF‐β2* was mainly expressed in CAFs (Figure 6C and D). Collectively, these results not only provide additional evidence to support the involvement of POSTN protein and TGF‐β signalling in the TME during LNM, but also pave the way for further investigations into the cellular interactions and diversity.

Subsequently, to further delve into tumour microenvironment presented in primary OSCC, we applied CopyKat (Copynumber Karyotyping of Tumors) to capture cancer cells.^103^ In total, we identified 5099 cancer cells within the subset of epithelial cells, which were further clustered into five clusters (C0‐C4) (Figures S8D and 6E). pLN+ OSCC were primarily composed of C0 and C1 clusters, whereas pLN– OSCC were mainly consisted of C2, C3, and C4 clusters (Figures S8E and 6E). Remarkably, the expression level of *TGFβI* was significantly higher in clusters C0 and C1 of pLN+ OSCC, designated as ‘TGFβI‐positive’ clusters (Figure 6F). Furthermore, to validate the cellular heterogeneity, we quantified cancer cell proportions in each cluster of pLN+ and pLN– OSCC. The data revealed that the ‘TGFβI‐positive’ cluster (C0 and C1) constituted over 80% of the pLN+ group, as compared to approximately 30% in the pLN– group. These results were consistent with our results based on the analysis of bulk tumour RNAseq data showing an increase in *TGFβI* expression in pLN+ OSCC (Figure S8F **)**. Subsequently, differential gene expression and gene set enrichment analyses showed genes related to cell–cell communication and ECM organisation were overexpressed in the C0 cluster, such as *DST* and the enrichment of EMT pathways (Figure 6G and H). *DST*, *BRIP1*, and *CENPP* were upregulated in the C1 cluster, leading to the enrichment of EMT and cell cycle‐related pathways, including MYC targets, E2F targets, and G2M checkpoint (Figure 6G and H). Moreover, both clusters exhibited higher expression levels of TGF‐β receptors *TGF‐βR1* and *TGF‐βR2*, indicating TGF‐β signalling was activated in cancer cells of pLN+ OSCC (Table S5). Taken together, our results based on the activation of both upstream genes and downstream receptors may indicate the TGF‐β signalling pathway was indeed activated in cancer cells within the pLN+ group.

Consequently, given that *TGF‐β1* and *TGF‐β2* genes are expressed in CAFs, we aimed to delineate cell–cell communication between CAFs and cancer cells since the cellular interaction might play a significant role in shaping the TME.^104^ Among these connections related to the TGFβ signalling pathway, we identified presence of a connection between the CAFs to C0 and C1 within the pLN+ OSCC, which is absent in the pLN– phenotype (Figure 6I and J). These results suggest that the activation of the TGF‐β pathway in cancer cells may be induced by the cytokines (TGF‐β1 and TGF‐β2) that are secreted by CAFs.

To further complement these findings, we employed spatial transcriptomics (ST) of five fresh‐frozen OSCC samples, including 2 pLN+ and 3 pLN– using the 10× Genomics Visium CytAssist platform. This approach preserved the 2D transcriptional information of cells, providing a spatial view of transcriptional heterogeneity within the TME.^105^, ^106^ Therefore, preceding to the snRNAseq findings, we subsequently picked up the top‐50 ranked DEG in each subcluster of cancer cells, and evaluated the signature of CAF and each subcluster in both pLN+ and pLN– samples. Accordingly, the results illustrated the pLN+ OSCC exhibited elevated expression on the signature CAF, C0, and C1 clusters, compared to the pLN– OSCC (Figure 6K and L). Subsequently, in order to gain more information about the localisation and interaction among these cells in the TME, we calculated the correlation analysis across these spatial spots. Not surprisingly, the pLN+ OSCC exhibited a significantly positive correlation among CAF, c0, and c1 clusters, whereas these correlations are significantly negative in pLN– OSCC (Figure 6M and N). These results suggest that CAF, C0, and C1 co‐localise in the TME of pLN+ OSCC, which further validated the crosstalk between CAF and ‘TGFβI‐positive’ cancer cells. Furthermore, we performed Gene Set Variation Analysis (GSVA) to estimate the enrichment of gene sets in specific tumours, revealing that the EMT pathway was significantly enriched in the pLN+ sample in contrast to the pLN– sample (Figure S8G and H). Collectively, these findings further validate that CAF could facilitate the invasiveness and metastasis of ‘TGFβI‐positive’ cancer cells via EMT during LNM in OSCC.

In summary, our single‐cell and spatial transcriptome analyses reveal that CAF‐secreted TGF‐β1/2 plays a pivotal role in promoting metastasis in pLN+ OSCC by activating EMT pathways. These insights underscore the significance of TGF‐β signalling in the TME and its potential to induce the lymph node dissemination in OSCC.

## 3 DISCUSSION

Oral squamous cell carcinoma (OSCC) remains a significant global health challenge, with over 370 000 new cases diagnosed annually.^107^ Early‐stage OSCC is typically managed with surgery, while radiation therapy, either alone or combined with systemic therapy, is also commonly employed.^108^ However, for advanced stages involving metastasis, systemic therapies such as chemotherapy, targeted therapy and immunotherapy become essential.^109^ Despite immunotherapy being a crucial component in the treatment of metastatic OSCC, its combination with curative radiotherapy or chemoradiotherapy has not consistently improved patient outcomes.^110^ Consequently, patients with lymph node metastases (LNM) face a significantly reduced prognosis and higher risks of distant metastases. Despite recent advances, the molecular mechanisms underlying early metastasis and the proteogenomic landscape of primary OSCC with LNM remain unclear, hindering the development of novel therapeutic targets. This study aimed to address this gap by conducting comprehensive multi‐omics and spatial analyses of primary OSCC with or without LNM, to uncover the molecular underpinnings of cancer cell dissemination and inform treatment strategies.

In this study, we have illustrated several critical pathways, gene, and proteins that related to the phenotype of OSCC with lymph node metastasis and suggested the underlying mechanism of disseminating to the lymph node may involve the cellular interactions between CAF and specific cancer cells, Our study provides important insights into the molecular mechanisms underlying OSCC with LNM, and lays a foundation for future research to delve into the functional role of the identified molecular signatures and pathways. Future innovative studies building upon our findings are encouraged to conduct deeper mechanistic exploration and uncover new therapeutic targets and strategies for managing OSCC.

One of the limitations of our study is the relatively small sample size and the imbalance in sample numbers between the two comparison groups. While we acknowledge that a larger and more balanced cohort would provide more robust statistical power, our findings still offer valuable insights into the molecular differences between pLN+ and pLN– OSCC. Future studies with expanded sample sizes and balanced group distributions are warranted to further validate and extend our observations. Another important consideration is that our study aimed to compare primary tumour samples from OSCC patients with and without lymph node metastasis. Although we did not include metastatic samples in our analysis, the focus on primary tumours allowed us to identify molecular signatures that may predispose to lymph node involvement. This approach is crucial for understanding the early events that drive metastasis and can inform the development of prognostic biomarkers and targeted therapies. However, future investigations incorporating both primary and metastatic samples would provide a more comprehensive view of the molecular landscape of OSCC progression.

Other than the findings obtained, this study is pioneering as it is the first to explore OSCC at the proteomic level. Advancements in genetic sequencing technology have revolutionised precision oncology, enabling the identification of mutations and alterations in cancer genomes. However, sequencing data alone often fails to provide a complete understanding of the functional properties of genome‐encoded proteins.^111^, ^112^ Quantitative proteomics has emerged as a promising approach to identifying disease biomarkers and molecular targets, enhancing our understanding of malignant transformation and therapeutic strategies.^113^, ^114^, ^115^, ^116^ This study breaks new ground by focusing on integrated analyses of bulk tumour sequencing data, particularly from a proteomic perspective, an area previously underexplored in OSCC studies. Our innovative proteomic layer‐based multi‐omics analysis of primary OSCC provided complementary insights beyond current genomic knowledge, paving the way for novel therapeutic interventions.

The extracellular matrix (ECM) plays a crucial role in maintaining tissue homeostasis, and its dysregulation is a hallmark of cancer progression.^117^ Our study revealed that ECM reorganisation in LNM is driven by the activation of the transforming growth factor‐β (TGF‐β) signalling pathway and cell cycle dysregulation. This interaction contributes to an immunosuppressive tumour microenvironment, correlating with poor prognosis for LNM patients. Our findings are consistent with previous studies showing that TGF‐β1‐associated ECM genes recruit cancer‐associated fibroblasts (CAFs), conferring immune evasion and resistance to cancer immunotherapies.^118^ Therefore, our study highlights the significance of ECM reorganisation, TGF‐β signalling activation, and cell cycle dysregulation in shaping the immunosuppressive microenvironment of LNM, presenting potential targets for novel therapeutic strategies.

*TGF‐βI* (Transforming growth factor β induced) is a gene responsive to the TGF‐β signalling pathway, induced by TGF‐β1/2, and localised to the ECM.^119^ Our transcriptomic and proteomic analyses revealed its upregulation in pLN+ OSCC, with enrichment in the TGF‐β and ECM remodelling pathways. Wang et al. found *TGF‐βI* elevation in late‐stage OSCC, indicating poor prognosis and potentially leading to an imbalanced immune response and OSCC progression.^120^ Additionally, *TGF‐βI* has been shown to enhance the efficacy of chemotherapy drugs like paclitaxel, cisplatin and gemcitabine in lung and ovarian cancers.^65^, ^121^ Thus, *TGF‐βI* could serve as a novel biomarker for treating OSCC with LNM.

The immune system plays a critical role in defending against cancer, making immunotherapy a rapidly evolving field. However, lymph node metastasis impairs anti‐tumour immunity, leading to immune suppression.^122^, ^123^ Single‐cell studies in head and neck squamous cell carcinoma (HNSCC) patients have shown a decrease in stem‐like CD8+ T cells and an increase in exhausted CD8+ T cells during LNM.^26^ Spatial transcriptomic analysis revealed that *CCXL12* expression leads to regulatory T‐cell infiltration and increased TGF‐β secretion, promoting a tumour immunosuppressive microenvironment.^124^ Our study builds on these findings, demonstrating that immunosuppressive signatures are exclusively present in pLN+ OSCC, providing further evidence that LNM induces an immunosuppressive microenvironment. This highlights the urgent need for a systematic exploration of this microenvironment and biomarkers to develop novel immunotherapies for metastatic disease.

Cell cycle dysregulation, a hallmark of cancer, leads to uncontrolled cell growth and tumour formation.^125^ Our transcriptomic analysis identified alterations in G2/M checkpoint genes in primary OSCC with LNM, which were negatively correlated with immune infiltration. This suggests that cell cycle dysregulation contributes to immune suppression during metastasis. Evidence from genomic and transcriptomic profiling indicates a link between immune evasion and cell cycle activity in cancer cells. Genetic amplification of *cyclin D1* (a CDK4/6 regulator) and *CDK4*, critical for cell cycle transitions, correlates with reduced efficacy of immune checkpoint blockade therapies.^126^ CDK4/6 inhibitors have shown promise in clinical trials, suggesting that targeting cell cycle proteins may effectively treat metastatic OSCC.^127^ The G2/M checkpoint has also emerged as a potential target, with inhibitors showing promise in treating glioblastoma.^128^ Thus, cell cycle inhibitors could represent novel therapeutic targets for metastatic OSCC.

POSTN (periostin), a matricellular protein involved in cell‐matrix interactions, is often overexpressed in cancers, promoting tumour growth, invasion and metastasis.^129^ POSTN activates PI3K/Akt and MAPK/ERK signalling pathways, regulates cell growth and metastasis, and shapes the tumour microenvironment.^130^, ^131^ It also modulates the immune response by stimulating immune cell recruitment and activation, facilitating tumourigenesis and invasion.^132^, ^133^ Our proteomic analysis identified POSTN as a pivotal protein in ECM remodelling, with elevated levels correlating with poor prognosis in LNM patients. Therefore, targeting POSTN could offer a promising therapeutic approach for OSCC patients.

Cancer‐associated fibroblasts (CAFs) play various roles in OSCC, including promoting cancer cell proliferation, migration, invasion and epithelial–mesenchymal transition (EMT).^134^ Previous studies have shown that cancer cell‐secreted molecules drive CAF activation in HNSCC, resulting in phenotypic changes and altered ECM production.^135^ Our single‐cell and spatial analyses identified that CAFs secrete TGF‐β1/2, enhancing TGF‐βR1/2 expression in cancer cell subclusters in pLN+ OSCC. This suggests that CAFs activate the TGF‐β pathway in cancer cells, facilitating communication and transformation between CAFs and cancer cells, which is exclusive to the pLN+ subtype. Therefore, targeting CAFs or their associated pathways could offer novel therapeutic interventions.

Given that cancer cell clusters in pLN+ OSCC are ‘*TGF‐βI* positive’ with enriched EMT functions, we speculate that TGF‐βI is involved in upregulating EMT by inducing the marker genes in these subclusters, such as COL17A1, ITGA6 and CDH13. Future studies should examine the interplay between TGF‐βI and these genes to better understand CAF and cancer cell crosstalk during OSCC metastasis. POSTN, identified in bulk proteogenomic analysis, may also play a critical role in these cell communications and microenvironment remodelling during cancer cell dissemination. Single‐cell and spatial analyses have shown that *POSTN+*CAFs are enriched in advanced non‐small cell lung cancer (NSCLC) tumours, associated with ECM remodelling, tumour invasion, and immune suppression.^136^ Therefore, we hypothesise that *POSTN+*CAFs are enriched in pLN+ OSCC and contribute to immune suppression, which warrants further exploration in future studies.

In summary, our integrated proteogenomic analysis has identified crucial pathways implicated in the tumourigenesis of primary OSCC with LNM and potential drivers of an ‘immune‐suppressive’ phenotype. This tumour microenvironment may be driven by augmented POSTN, ECM remodelling, TGF‐β pathway activation, and cell cycle control disruption. In particular, the cytokines in the TGF‐β pathway may be induced by CAFs, which in turn activate this pathway and facilitate CAF and cancer cell communication for promoting cancer cell proliferation and metastasis. These findings enhance our understanding of OSCC progression and highlight viable targets for developing novel therapeutic interventions. Further studies should explore the subtype of CAFs and identify key factors in the TGF‐βI/EMT axis for OSCC progression and metastasis.

## 4 METHODS

### 4.1 Biological sample collection and processing

Tumour tissue and matched blood samples were collected from oral squamous cell carcinoma (OSCC) patients diagnosed at Oral and Maxillofacial Surgery Department, Queen Mary Hospital, Hong Kong, between Jul 2016 and Dec 2019. Patients consented to research use of their tissues, as approved by the Institutional Review Board of the University of Hong Kong/Hospital Authority Hong Kong West Cluster (IRB Reference Number: UW 15–239). Clinicopathological and follow‐up data were retrospectively gathered from hospital records. Fresh tumour tissues were divided into two parts: one for formalin‐fixed paraffin‐embedded (FFPE) processing and the other quickly frozen for storage. Fresh frozen blood samples served as normal controls. Both fresh frozen tumour and blood samples were stored at −80°C until extraction, while FFPE samples were kept at room temperature for staining.

### 4.2 DNA and RNA extraction

#### 4.2.1 Germline DNA extraction

Germline DNA was extracted from frozen blood samples using the TIANamp Genomic DNA kit (Catalogue #: DP304) following the manufacturer's instructions.

#### 4.2.2 Tumour DNA extraction

Tumour DNA was extracted from fresh frozen tumour samples using cetyltrimethylammonium bromide (CTAB). The tissue block was ground with liquid nitrogen, and 50 mg was transferred to a 2.0 mL centrifuge tube containing 1 mL of CTAB lysis buffer. The mixture was incubated at 65°C with occasional mixing until fully lysed. The lysate was then centrifuged, and the supernatant was extracted with phenol (pH 8.0): chloroform: isoamyl alcohol (25:24:1), followed by chloroform: isoamyl alcohol (24:1). DNA was precipitated with isopropanol at –20°C, centrifuged, washed twice with 75% ethanol, and air‐dried. The DNA was dissolved in ddH_2_O, incubated at 55–60°C if necessary, and treated with RNase A at 37°C for 15 min.

#### 4.2.3 Tumour RNA extraction

Tumour RNA was extracted from fresh frozen tumour tissue using Trizol. The tissue block was ground with liquid nitrogen, and 50–100 mg was transferred to a 2.0 mL centrifuge tube containing 1 mL of Trizol. Chloroform (1/5 volume) was added, and the mixture was shaken vigorously, incubated at room temperature for 2–3 min, and centrifuged at 12 000 rpm at 4°C for 10–15 min. The upper aqueous phase was transferred to a new tube, mixed with an equal volume of isopropanol, and incubated at room temperature for 10 min. After centrifugation at 12 000 rpm at 4°C for 10 min, the supernatant was discarded, and the RNA pellet was washed with 75% ethanol prepared with DEPC water. The pellet was air‐dried, dissolved in RNase‐free water (DEPC water), and stored at –80°C.

### 4.3 Whole‐exome sequencing

Exome sequences were enriched from .4 µg of genomic DNA using the Agilent SureSelect Human All Exon V6 (Catalogue #: 5190–8864) according to the manufacturer's protocol. Genomic DNA was fragmented to an average size of 180–280 bp using the Covaris S220. Overhangs were converted to blunt ends via exonuclease and polymerase activities. DNA fragments were end‐repaired, phosphorylated, A‐tailed, and ligated with paired‐end adaptors at the 3′ ends. Adapter‐ligated DNA fragments were enriched through PCR. Libraries were hybridised with biotin‐labelled probes and captured using magnetic beads with streptomycin. Captured libraries were further enriched via PCR to add index tags for sequencing preparation. Libraries were purified using the AMPure XP system (Beverly, USA), analysed for size distribution by the Agilent 5400 system, and quantified by QPCR (1.5 nM). Qualified libraries were pooled and sequenced on Illumina platforms using the PE150 strategy at CHI BIOTECH CO., LTD.

### 4.4 RNA sequencing

Total RNA was used to generate sequencing libraries using the NEB Next Ultra RNA Library Prep Kit for Illumina (Catalogue #: E7530L). mRNA was purified from total RNA using poly‐T oligo‐attached magnetic beads. Fragmentation was performed with divalent cations under elevated temperatures. First‐strand cDNA synthesis was conducted using random hexamer primers and M‐MuLV Reverse Transcriptase (RNase H). Second‐strand cDNA synthesis was performed using DNA Polymerase I and RNase H. Overhangs were converted to blunt ends, and NEB Next Adaptor with a hairpin loop structure was ligated to the adenylated 3′ ends of DNA fragments. Library fragments were purified to select 370–420 bp cDNA fragments using the AMPure XP system. USER Enzyme was used for size selection, followed by PCR with Phusion High‐Fidelity DNA polymerase, Universal PCR primers, and Index (X) Primer. PCR products were purified, and library quality was assessed using the Agilent 5400 system and quantified by QPCR (1.5 nM). Qualified libraries were pooled and sequenced on Illumina platforms using the PE150 strategy at CHI BIOTECH CO., LTD.

### 4.5 Computational pipelines

All pipelines were developed according to National Cancer Institute sequencing guidelines. Tools from the GATK 4 suite were used for data processing.

### 4.6 Alignment and preprocessing

WES data preprocessing followed GATK Best Practices using GATK 4.0. Fastq files underwent quality control with ‘FastQC’. Sequencing adapters were removed with ‘Trimmomatic’. Fastq files were aligned to the hg38 genome using ‘BWA MEM’, and attributes were restored using ‘MergeBamAlignment’. PCR and optical duplicates were marked with ‘MarkDuplicates’. Base recalibration was performed using ‘BaseRecalibrator’ and ‘ApplyBQSR’. Coverage statistics were gathered using ‘CollectHsMetrics’. Alignment quality control was performed with ‘ValidateSamFile’ and inspected using ‘MultiQC’.

RNA data preprocessing followed the mRNA analysis pipeline from the National Cancer Institute. Raw fastq files underwent quality control with ‘FastQC’. Bases failing quality control were trimmed using ‘Trimmomatic’. Fastq files were aligned to the hg38 genome using ‘ Hisat2’ to generate BAM files. Missing values were imputed using the impute.knn package, with imputation on the genes present in at least 50% of samples.

### 4.7 WES variant detection

Variant detection followed GATK Best Practices using GATK4. Germline variants were called from control samples using Mutect2 in artefact detection mode and pooled into a cohort‐wide panel of normal samples. Somatic variants were called from tumour samples with matched normal controls using Mutect2, with parameters including the matched normal sample, reference fasta file, panel of normal, and gnomAD germline resources. Cross‐sample contamination was evaluated using ‘GetPileupSummaries’ and ‘CalculateContamination’. Read orientation artefacts were assessed using ‘Collect‐F1R2Counts’ and ‘LearnReadOrientationModel’. Additional filters were applied using ‘FilterMutectCalls’.

### 4.8 WES variant post‐processing

BCFTools was used to normalise, sort, and index variants. A consensus VCF was generated, removing duplicate variants. The VCF file was annotated using ANNOVAR with COSMIC, dbSNP, refGene data source.

### 4.9 Mutational burden

The mutational burden was calculated as the number of mutations per Mb sequenced. A minimum coverage threshold of 30x was required for each base. We regarded 38 Mb as the estimate of the exome size.^137^

### 4.10 Tumour heterogeneity and MATH

Heterogeneity was inferred by clustering VAF in primary OSCC samples. The median absolute deviation (MAD) of mutant‐allele fractions (MAF) was calculated for all tumours. MATH score, representing intratumour heterogeneity (ITH), was calculated as the percentage ratio of MAD to the median MAF value among the tumour's mutated loci.

### 4.11 Unique and shared mutations

Mutations in pLN+ samples were compared to matched pLN– samples. Shared mutations were defined as identical mutations at the same chromosomal positions leading to the same variants in the same genes. Unique mutations were those not shared between the groups.

### 4.12 Mutational signatures and oncoplots

COSMIC mutational signatures were determined from mutations in primary OSCC samples. A mutation matrix was formed and decomposed into multiple signatures using NMF, compared to known COSMIC signatures based on cosine similarity. Differentially mutated genes were identified using a Fisher test and visualised with oncoplots.

### 4.13 Copy number segmentation and calling

Copy number identification was conducted via an open‐source software called CNVkit with default settings, which is a tool kit to infer and visualise copy number from targeted DNA sequencing data.^138^ GISTIC2.0 identified SCNAs, with segmented copy numbers deconstructed using the ‘Ziggurat Deconstruction’ algorithm and significant SCNAs determined using the ‘Arbitrated Peel‐off’ algorithm.

### 4.14 Pathway mutation perturbation level measurement

Pathway activation was identified by mapping mutations to genes and applying criteria such as mutation frequency. Gene mutation perturbation scores (GMPscore) and pathway mutation perturbation scores (PMPscore) were calculated iteratively.^48^

### 4.15 Differential gene expression analysis of RNAseq data

Following alignment, BAM files were processed through the RNA Expression Workflow to determine RNA expression levels. Reads were mapped to each gene using Hisat2.^139^ Transcripts were assembled using Stringtie, and the number of reads mapped to each gene was normalised using ‘DESeq2’, which employs a negative binomial distribution. DESeq2 provided base means across samples, log2 fold changes, standard errors, test statistics, *p*‐values, and adjusted *p*‐values. Significant genes were visualised using a ‘Volcano Plot’.

### 4.16 Gene set enrichment analysis

Pathway analysis was conducted using ‘fgsea’, a fast preranked GSEA. Ranked significant genes from ‘DESeq2’ and the Reactome pathway dataset (c2.cp.Reactome.v7.4) were used as inputs for ‘fgsea’, generating outputs including pathway names, enrichment scores, normalised enrichment scores, and *p*‐values.

### 4.17 Immune cell abundance analysis

Relative immune cell fractions for downstream neoantigen analysis were determined using the ‘MCPcounter’ R package. ESTIMATE relative immune cell analysis was conducted using the ‘Estimate’ R package.^75^ Gene expression data was used in CIBERSORTx to estimate the abundances of member cell types in a mixed cell population.

### 4.18 Mann–Whitney–Wilcoxon Gene‐Set Test (MWW‐GST)

To evaluate pathway activity, we calculated the normalised enrichment score of all pathways among four gene sets (GO‐BP, KEGG, Hallmark, and immunologic signature gene set) using MWW‐GST with the ranked list of DEGs. The MWW test statistic normalisation provided the Normalised Enrichment Score (NES), an estimate of probability.

### 4.19 Protein extraction

Fresh‐frozen tumour samples were ground with liquid nitrogen, and the powder was transferred to a 1.5 mL centrifuge tube. Samples were sonicated in a lysis buffer (8 M urea with 1 mM PMSF and 2 mM EDTA), and debris was removed by centrifugation at 15 000 × *g* at 4°C for 10 min. Protein concentration was determined with a BCA kit. Equal amounts of proteins were digested with trypsin. The mix was reduced with 10 mM DTT for 45 min at 37°C and alkylated with 50 mM iodoacetamide for 15 min in the dark. Proteins were precipitated with chilled acetone at –20°C for 2 h, air‐dried, resuspended in 25 mM ammonium bicarbonate and digested overnight at 37°C with trypsin. Peptides were desalted using a C18 Cartridge, dried and redissolved in .1% formic acid.

### 4.20 LC‐MS/MS detection

Liquid chromatography (LC) was performed on a nanoElute UHPLC. Approximately 200 ng peptides were separated over 60 min at .3 µL/min on a reverse‐phase C18 column with an integrated CaptiveSpray Emitter. The temperature was maintained at 50°C. Mobile phases A and B were .1% formic acid in water and .1% formic acid in HPLC‐grade acetonitrile (ACN), respectively. Mobile phase B was increased from 2% to 22% over 45 min, to 35% over the next 5 min, to 80% over the next 5 min, and held at 80% for 5 min. The LC was coupled online to a timsTOF Pro2 operated in Data‐Dependent Parallel Accumulation‐Serial Fragmentation (PASEF) mode with 10 PASEF MS/MS frames in one complete frame. The capillary voltage was set to 1400 V, and MS/MS spectra were acquired from 100 to 1700 m/z.

### 4.21 MS data analysis

MS raw data were analysed using DIA‐NN (v1.8.1) with a library‐free method. The Homo sapiens SwissProt database (20425 entries) was used to create a spectral library with neural network algorithms. MBR was employed to create and reanalyse a spectral library from DIA data. FDR was adjusted to < 1% at both protein and precursor ion levels. Identifications were used for further quantification analysis. Missing values were imputed using the impute.knn package, with imputation on the genes present in at least 50% of samples.

### 4.22 Weighted correlation network analysis (WGCNA)

Quantifiable proteins were analysed with WGCNA to construct a protein co‐expression network using the R package ‘WGCNA’.^88^ Scale‐free *R* ^2^ = .8 was used for consistency with scale‐free characteristics. The adjacency matrix was transformed into a topological overlap matrix (TOM) to reduce noise and spurious correlation. Network construction and module identification were based on TOM similarity. Parameters were set as follows: soft‐threshold power (β) = 12, ‘cutreeDynamic’ function, minModuleSize = 20. Functional annotation of each module was performed using the compareCluster subprogram of the R package ‘clusterProfiler’, DAVID, and STRING.^140^, ^141^, ^142^ Annotation gene sets for compareCluster analysis were downloaded from MSigDB (Hallmark gene sets, KEGG pathway database, and biological process from Gene Ontology).^140^ The biological function of each module was summarised by the most significant enriched pathways (adjusted *p*  <  .05). Force‐directed layout visualisation of the 31 functional modules was created using the R package ‘igraph’. The protein co‐expression network was visualised using Cytoscape v3.6.0.^143^

### 4.23 Correlation between module scores and clinical features

Statistical analysis of the correlation between module scores and clinical features included:

1. Prognosis evaluation for OS analysis: Patients were segregated into two groups based on the median module score. *p* values were calculated using the log‐rank test. Modules were categorised as favourable (*p*  < .05, HR  <  1), unfavourable (*p*  <  .05, HR  >  1), or not significant (*p* ≥ .05).
2. Continuous factor analysis: Spearman's correlation explored relationships between module scores and continuous clinical features (e.g., age). Modules were classified as negative (*p*  <  .05, *r*  <  0), positive (*p*  <  .05, *r*  >  0), or not significant (*p* ≥ .05).
3. Categorical factor analysis: For binary factors (e.g., gender, alcohol consumption), *p* values were computed using the Mann–Whitney U‐test. For factors with more than two categories (e.g., stage, grade), *p* values were derived using ANOVA. Significance was defined as *p*  < .05.

### 4.24 Survival analysis

Kaplan–Meier analysis explored survival differences associated with lymph node metastasis group, ME expression level, and POSTN expression level. Statistical significance was assessed using Kaplan–Meier plots, log‐rank tests, and Cox proportional hazards regression via the survminer (version 0.4.9) and survival (version 3.2‐13) R packages.

### 4.25 Illumina infinium MethylationEPIC BEADCHIP and data processing

Genomic DNA concentration and integrity were assessed using a NanoDrop 2000 spectrophotometer and agarose gel electrophoresis. DNA was bisulphite‐treated using the Zymo Research EZ DNA Methylation‐Glod Kits. Bisulphite‐converted DNA was analysed on an Illumina Infinium MethylationEPIC v2.0 (935K) BeadChip and scanned using Illumina iSCAN. Idat files were preprocessed with the ChAMP (version 2.12.4) package in R and normalised using the BMIQ method.^144^ Statistical differences in continuous variables were compared by *t*‐test.

### 4.26 Differential methylation analysis

Differential methylation analysis was performed between 20 pLN+ and 5 pLN– tumour samples. Probes containing SNPs, probes in chromosome X, and probes with more than 10% missing values were excluded. The Wilcoxon rank‐sum test determined differentially methylated CpGs (DMPs), with *p*‐values adjusted by the FDR method. DMPs were reported if the mean methylation difference was > .2 with an FDR of 5%.

### 4.27 Differentially methylated region (DMR) analysis

DMRs were identified separately for the two groups and combined using meta‐analysis with the ‘dmrf’ package in R and Rex (version 3.6.0).^145^ Methylation levels of each CpG site were transformed using inverse normal transformation to ensure robustness against outliers and normal distribution assumptions. Regions with a maximum distance of 500 bp between consecutive features and at least two significant probes (*p* < .05) were identified. DMRs were evaluated using a Bonferroni adjusted significance level of .05. Annotation was performed using the ENSEMBL_MART_ENSEMBL BioMart database and the hsapiens_gene_ensembl database in the Ensembl genome browser (version: GRCh37).

### 4.28 Gene set analysis (GSA)

Gene ontology (GO) terms were identified using significant CpGs and DMRs. Terms with at least five CpG sites were used to create a gene set, tested using a .05 FDR‐adjusted significance level. GSA was performed using the ‘missMethyl’ package in R.^145^, ^146^

### 4.29 mRNA–protein correlation analysis

A total of 8375 genes or proteins with less than 50% missing values were analysed for gene‐wise and sample‐wise mRNA and protein correlations. Spearman's correlation coefficient and corresponding *p* value were calculated for each mRNA–protein pair across pLN+ and pLN– tumours and individual samples using the cor.test function in R. Adjusted *p* values were calculated using the Benjamini–Hochberg (BH) procedure, with a cut‐off of .01 for statistical significance.

### 4.30 Prognostic biomarker analysis

To identify potential protein prognostic biomarkers, four criteria were used:

1. Proteins must be quantified in all samples.
2. The correlation c oefficient between mRNA and protein expression should be >.7.
3. Candidate proteins should be differentially expressed between pLN+ and pLN– OSCC with adjusted *p* value < .01 (Wilcoxon signed‐rank test, BH adjusted) and fold change > 1 at both mRNA and protein levels.
4. Kaplan–Meier curve with log‐rank test visualised survival differences, and Cox proportional hazard model evaluated the hazard ratio (HR) for each protein. Candidate proteins should significantly correlate with overall survival (log‐rank *p* value < .01, and HR (high/low) > 2 for upregulated or < .5 for downregulated proteins).

### 4.31 Immune scores correlation analysis

To identify potential drivers of immunosuppression, ESTIMATE immune scores were correlated with mRNA–protein data using Spearman's correlation analysis. We performed GSEA for KEGG pathways using signed –log10 *p* values.^147^ Gene set‐based scores were the mean protein expression of all genes in that set.

### 4.32 Single nucleus isolation and sequencing

Nuclei were isolated using the Shbio Nuclei Isolation Kit (SHBIO, #52009‐10, China) and counted with a cell counter (Thermo Fisher). Using a Chromium Single Cell 3′ Library and Gel Bead Kit v3 (10X Genomics), nuclei were loaded onto a Chromium Single Cell Processor (10X Genomics) to barcode RNA from single nuclei. Sequencing libraries were constructed according to the manufacturer's instructions (10× Genomics) and sequenced on a NovaSeq 6000 system (Illumina, 20012866).^148^

### 4.33 snRNA‐seq data processing

Reads were processed using the Cell Ranger 3.0.1 pipeline with default and recommended parameters.^149^ Gene‐Barcode matrices were generated by counting UMIs and filtering non‐cell associated barcodes. The gene‐barcode matrix containing barcoded cells and gene expression counts was imported into Seurat_4.1.3 R toolkit for quality control and downstream analysis of single‐cell RNAseq data.^150^

### 4.34 snRNAseq clustering analysis

Cluster analysis of single‐cell count matrices was performed using the R package ‘Seurat’ (v4.1.3).^151^ Normalisation and scaling were performed after filtering using the ‘NormalizationData’ and ‘ScaleData’ functions with default parameters. Principal components for highly variable genes were calculated using ‘RunPCA’. After quality control, removal of batch effects, and data integration from 4 samples (2 pLN+ and 2 pLN–), 22 433 cells were used in downstream analysis. Clusters were identified using ‘FindClusters’ with a .5 resolution. Uniform manifold approximation and projection (UMAP) visualised clusters in a reduced 2D space. Cluster markers were identified using ‘FindAllMarkers’, and cell types were assigned using cluster markers and CellMarker 2.0.^152^

### 4.35 Cancer cell prediction

The CopyKAT package predicted cancer cells from epithelial cells.^103^ Cells from non‐epithelia war regarded as reference, while cells with considerable aneuploidy mutations were considered cancer cells. Each sample was calculated individually and all cells with aneuploidy mutations were annotated for downstream analysis as a new cell type.

### 4.36 Cell–cell communication analysis

The R package Cellchat (version 2.1.2) inferred interaction mechanisms among tumour microenvironment (TME) components across pLN+ and pLN– tumour tissues.^153^ The ‘anadata’ dataset was transformed to generate a new Cellchat object. Functions such as ‘compareInteractions’, ‘netVisual_heatmap’, ‘netAnalysis_signalingRole_scatter’, and ‘rankNet’ analysed and compared interaction numbers, strengths, and information flow of signalling pathways or ligand‐receptor pairs among tissues. Interaction numbers and strengths among different cellular components within tissues were also investigated.

### 4.37 Spatial sequencing library preparation

Samples with RNA quality control (RIN > 4) were used for spatial transcriptomic construction and sequencing. 10‐micron thick sections were mounted onto glass slides, fixed in ice‐cold methanol, stained with hematoxylin and eosin, and scanned under a microscope (Keyence, Itasca, IL, USA). The stained slide was incubated with a Human whole transcriptome probe panel and transferred to Cytassist (10× Genomics). A human whole transcriptome probe panel (10×) consisting of three pairs of specific probes for most genes was hybridised to RNA. Probe pairs were ligated, forming single‐stranded ligation products. Samples were treated with RNase and permeabilised to release ligation products. Poly‐A portions of products were captured by poly(dT) regions of capture probes on the Visium slide, including an Illumina Read 1, spatial barcode, and unique molecular identifier (UMI). Probes were elongated to generate spatially barcoded ligated probe products, detached from the slide, indexed through Sample Index PCR, and sequenced. Visium Spatial Gene Expression libraries consisted of Illumina paired‐end sequences flanked with P5/P7. The 16‐bp Spatial Barcode and 12‐bp UMI were encoded in Read 1, while Read 2S sequenced the ligated probe insert.

### 4.38 Signature expression analysis of spatial spots

We selected the top 50 ranked DEGs in each cluster as the signatures, then calculated the cluster scores for feature expression programs in spatially single‐cell level via addmodulescore function in ‘Seurat’ R package (version 4.4.0), with the default settings.

### 4.39 Gene set variation analysis (GSVA)

Pathway activities of tumour cluster spots were quantified using GSVA, implemented in the GSVA package.^154^ The log‐transformed normalisation expression matrix of tumour spots was input into the ‘gsva’ function with default parameters. A set of 50 cancer hallmark signatures was used for analysis.

## AUTHOR CONTRIBUTIONS

G.Z. designed the study and provided financial and administrative support. Y.L. contributed to data interpretation and manuscript editing. G.Z. and Y.S. jointly supervised the study. J.P. provided study patients’ information and contributed to clinical data interpretation. Y.L. and Z.Y. contributed to data analysis and data interpretation. Y.L., J.Z. and U.K. participated in collecting clinical samples and data. All authors involved in writing the manuscript and final approval of the manuscript.

## FUNDING

This research was supported by the Oral Health Research and Innovation Fund (the Faculty of Dentistry at the University of Hong Kong) (G.Z.); Collaborative Research Fund (No. C7015‐23GF), General Research Fund (No. 17117523), and Seed Fund for Collaborative Research (No. 2307102377), Hong Kong (Y.S.).

## CONFLICT OF INTEREST STATEMENT

The authors declare no competing interests.

## ETHICS STATEMENT

This study was approved by the Institutional Review Board of the University of Hong Kong/Hospital Authority Hong Kong West Cluster (IRB Reference Number: UW 15–239). Informed consent was obtained from all patients for the research use of their tissues.

## CODE AVAILABILITY

We applied no custom code or mathematical algorithm.

## Supporting information



Sample processing workflow for this study. A cohort of 50 patients was initially included, from which a subset of 41 tumour samples and corresponding blood controls underwent whole‐exome sequencing (WES). Additionally, 33 tumours were subjected to bulk tumour RNA sequencing, while 25 were analysed using DNA methylation arrays. Protein extraction and quality control were performed on 24 fresh frozen tumour samples, which were subsequently subjected to 4D‐microDIA proteomics sequencing. Spatial transcriptomic analysis was performed on 5 fresh frozen samples, and single‐nucleus RNA sequencing (snRNAseq) was performed on 4 samples. Kaplan–Meier survival curves comparing overall survival (OS) between OSCC patients with lymph node metastasis and those without lymph node metastasis in the QMH cohort (B) and the TCGA cohort (C).
**Figure S2. Supplemental gene mutation patterns**.
(A) Box plots comparing mutational burden between primary OSCC with and without lymph node metastasis in the QMH cohort. *p*‐Value calculated using Wilcoxon tests. (B) Box plots comparing mutant‐allele tumour heterogeneity between primary OSCC with and without lymph node metastasis in the QMH cohort. *p*‐Value calculated using Wilcoxon tests. Co‐Oncoplots showing shared and unique mutated genes in pLN– (C) and pLN+ (D) tumour samples in the TCGA cohort. Oncoplots showing individual mutated gene patterns in pLN– (E) and pLN+ (F) tumour samples in the TCGA cohort. (G) Box plots comparing COSMIC signatures between primary OSCC with and without lymph node metastasis in the QMH cohort.
**Figure S3. Landscape of somatic copy number alterations in pLN– and pLN+ tumour samples**.
Plots showing genomic locations and calculated *q*‐values for aberrant regions as determined by the Genomic Identification of Significant Targets in Cancer (GISTIC) method for tumour samples in the QMH cohort. (A, C) Amplification and depletion regions reported for pLN–. (B, D) Amplification and depletion regions reported for pLN+. The genome is oriented vertically from top to bottom, with GISTIC scores at each locus plotted from left to right at the top and q‐values plotted on a log scale at the bottom. The green line represents the significance threshold (*q*‐value = .25).
**Figure S4. Differentially expressed genes (DEG) and CIBERSORTx findings from bulk tumour RNAseq**.
(A) Volcano plot showing differentially expressed genes between pLN– and pLN+ groups. (B) Box plot representing CIBERSORTx scores comparison between primary OSCC with and without lymph node metastasis.
**Figure S5. Proteomic analysis revealing differentially expressed proteins (DEP), GSEA, and construction of protein co‐expression network**.
(A) Volcano plot showing differentially expressed proteins between pLN– and pLN+ groups of 24 patients. (B) Gene Set Enrichment Analysis (GSEA) plots of the top 20 pathways (ranked by adj. *p* values) based on the Reactome collection from MSigDB. The red bar towards the right indicates pathways enriched in primary OSCC with lymph node metastasis, and the purple bar towards the left indicates pathways enriched in primary OSCC without lymph node metastasis. Pathways enriched in pLN+ OSCC related to ECM remodelling are highlighted in red. Analysis of the scale‐free fit index (C) and the mean connectivity (D) for determining soft‐thresholding powers. (E) Hierarchical clustering dendrogram of proteins in different modules. (F) Protein co‐expression network. Nodes are colour‐coded according to module membership. Functional enrichment in significantly dysregulated modules in the pLN+ group (G) and pLN– group (H). Colours represent the relative ratios of significant genes within each pathway, circle size represents the adjusted *p*‐values of each program in modules, and the x‐axis represents the individual module number.
**Figure S6. DNA methylation landscape of primary OSCC tumours**.
(A) Distribution of differentially methylated probes (DMPs) in different regions related to CpG islands, including islands, CpG shores, and CpG shelves, and gene regions (TSS1500, TSS200, 5′ UTRs, first exons, gene bodies and 3′ UTRs). (B) Heatmap showing the methylation probe location of CpG islands, shores, and shelves. (C) Differentially methylated regions covering the *EGFR* gene on chromosome 7. (D) GO‐BP pathway analysis of DMPs.
**Figure S7. Distribution of Spearman's correlation coefficients**.
(A) Distribution of Spearman's correlation coefficients between mRNA and protein log2‐fold changes of individual genes across patients. (B) Molecular network showing alterations in the pLN+ OSCCs on genomic, transcriptomic, proteomic, and epigenetic levels. Genes identified in the distinct platforms are highlighted in different colors.
**Figure S8. UMAP plot of cells distinguished by groups**.
(A) UMAP plot of the single‐cell profile coloured by pLN+ and pLN– groups. (**B)** UMAP plot of the single‐cell profile coloured by 10 clusters. (**C)** UMAP atlas showing the expression levels of POSTN. (D) UMAP plot showing CopyKat‐screened cancer cells with extensive genome‐wide copy number aberrations (aneuploidy). (E) UMAP plot of the cancer‐cell profile coloured by pLN+ and pLN– groups. (F) Cell abundance analysis showing the proportion of each cluster in pLN+ and pLN– OSCC. Spatial spots showing the enrichment level of EMT pathway in pLN– **(G)** and pLN+ OSCC **(H)**.

Supporting Information

## Data Availability

All processed WES, RNAseq, 4D‐microDIA proteomics, DNA methylation array, snRNAseq and spatial transcriptomic data have been deposited in the Figshare. Access to the data can be requested by the Figshare website via the private link (https://figshare.com/s/63746bc3df131dd7f212). The raw data of RNAseq has been submitted to GEO (GSE289930).
